# ERG-deficient endothelium identifies IL-8/CXCR2 axis as a therapeutic target for resolving neutrophilic lung vascular injury

**DOI:** 10.1172/jci.insight.195989

**Published:** 2026-03-05

**Authors:** Vigneshwaran Vellingiri, Vijay Avin Balaji Ragunathrao, Jagdish Chandra Joshi, Md Zahid Akhter, Mumtaz Anwar, Somenath Banerjee, Sayanti Datta, Viktor Pinneker, Steven Dudek, Yoshikazu Tsukasaki, Sandra Pinho, Dolly Mehta

**Affiliations:** 1 Department of Pharmacology and Regenerative Medicine, and; 2 Division of Pulmonary, Critical Care, Sleep and Allergy, University of Illinois Chicago, College of Medicine, Chicago, Illinois, USA.

**Keywords:** Inflammation, Pulmonology, Vascular biology, Endothelial cells, Innate immunity

## Abstract

Aberrant polymorphonuclear neutrophil (PMN) accumulation in tissues induces chronic vascular diseases. Endothelial cells (ECs) regulate the access of PMNs into the tissue from the blood. However, the mechanisms that prevent PMNs from being activated and accumulating in the tissue, a hallmark of acute lung injury (ALI), remain elusive. We demonstrate that conditional deletion of *Erg* in ECs spontaneously alters the PMN transcriptome, which is enriched with genes that induce PMN recruitment, adhesion, activation, and “do not eat me” signals due to impaired synthesis of the deubiquitinase A20. Decreased A20 levels, in turn, activated the transcription factor NF-κB and the secretion of MIP2α (human homolog of IL-8) in ECs. EC-secreted MIP2α/IL-8 engaged the CXCR2 cascade on PMNs, leading to their activation and inflammatory injury. These findings were recapitulated in the lungs and blood of PMNs from patients dying of ALI. Overexpression of the *A20* gene in ECs or pharmacological inhibition of CXCR2 on PMNs in *iEC-Erg^–/–^* mice rescued EC control of PMNs and tissue homeostasis, and enhanced mouse survival after pneumonia. Thus, the EC/Erg/A20 axis regulates PMN accumulation and hyperactivation in the lungs by inhibiting EC-mediated IL-8 activation of PMN CXCR2, thereby providing a potential target for neutrophilic inflammatory vascular diseases.

## Abstract 2

Endothelial loss of ERG subverts PMN transcriptome towards inflammatory lineage via IL8–CXCR2. CXCR2 blockade with Reparixin reduces inflammation, neutrophil infiltration, and improves pneumonia survival.

## Graphical abstract

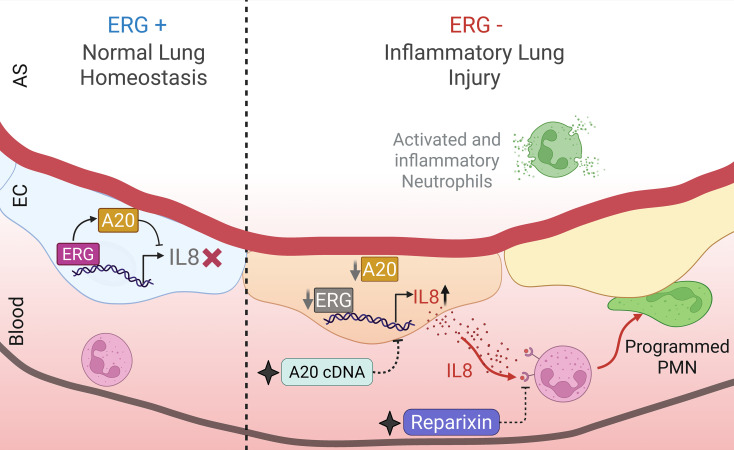

## Introduction

Polymorphonuclear neutrophils (PMNs) are the most abundant white blood cells (WBCs) and the first responders to be recruited from circulation to the tissues following environmental and blood-borne infections (1, 2). These cells eradicate infections by generating reactive oxygen species (ROS), cytotoxic granules, extracellular trap formation, and phagocytosis (3–6). PMNs are short-lived WBCs but can accumulate at affected sites in adequate numbers, such as in the lungs, due to enhanced expression of the genes promoting their survival and inflammatory activities (7, 8). The vascular endothelium is the first point of entry for PMNs to breach and cause tissue injury (9). The lung is one of the most vascularized organs in the body and is constantly exposed to blood-borne and inhaled pathogens, as well as other environmental stimuli. Neutrophilic inflammation and hyperpermeable lung microvasculature are consequential to lethal acute lung injury (ALI) and acute respiratory distress syndrome (ARDS) (10–12) due to a lack of therapeutic options (13). However, mechanisms that prevent PMN accumulation and activation in the tissue to protect against an excessive inflammatory response and injury are elusive (2, 14).

The ETS-related gene (*Erg*), a transcription factor expressed in ECs, regulates the expression of several genes as a super-enhancer (15–18), including *IL8* (18–20). MIP2α (mouse homolog of human IL-8) is the predominant chemokine activating CXCR2 on PMNs (21–23). *Erg* is known for regulating various endothelial functions, including vascular development and stabilization (24). EC-specific deletion of *Erg* is embryonically lethal due to defective vasculature formation (25). Conditional inactivation of *Erg* in ECs leads to impaired vascular development, induced vascular leak, and increased susceptibility to developing fibrosis during aging (26–31). Whether *Erg* activity in ECs influences PMN extravasation and activation in the lung circulation is not known. Understanding the *Erg* regulation of PMNs’ function in the lungs is therefore critical to improving treatment options for ALI/ARDS and other chronic inflammatory diseases.

Here, we identified a crucial role of *Erg* in controlling PMN infiltration and activation in the lungs at homeostasis and injury. We demonstrated that *Erg* prevents PMN recruitment, accumulation, and activation in the lungs by synthesizing the deubiquitinase A20. EC A20 suppresses NF-κB activity and IL-8/MIP2α secretion from ECs into the circulation, thereby inhibiting the engagement of PMN CXCR2 signaling. These studies identified a previously unknown role of *Erg* in ECs in suppressing neutrophilic injury in vivo by synthesizing A20, which can be targeted to resolve inflammatory disease states, such as ALI.

## Results

### EC Erg suppresses PMN infiltration and activation, thereby maintaining tissue homeostasis at a steady state.

**Figure 1:**
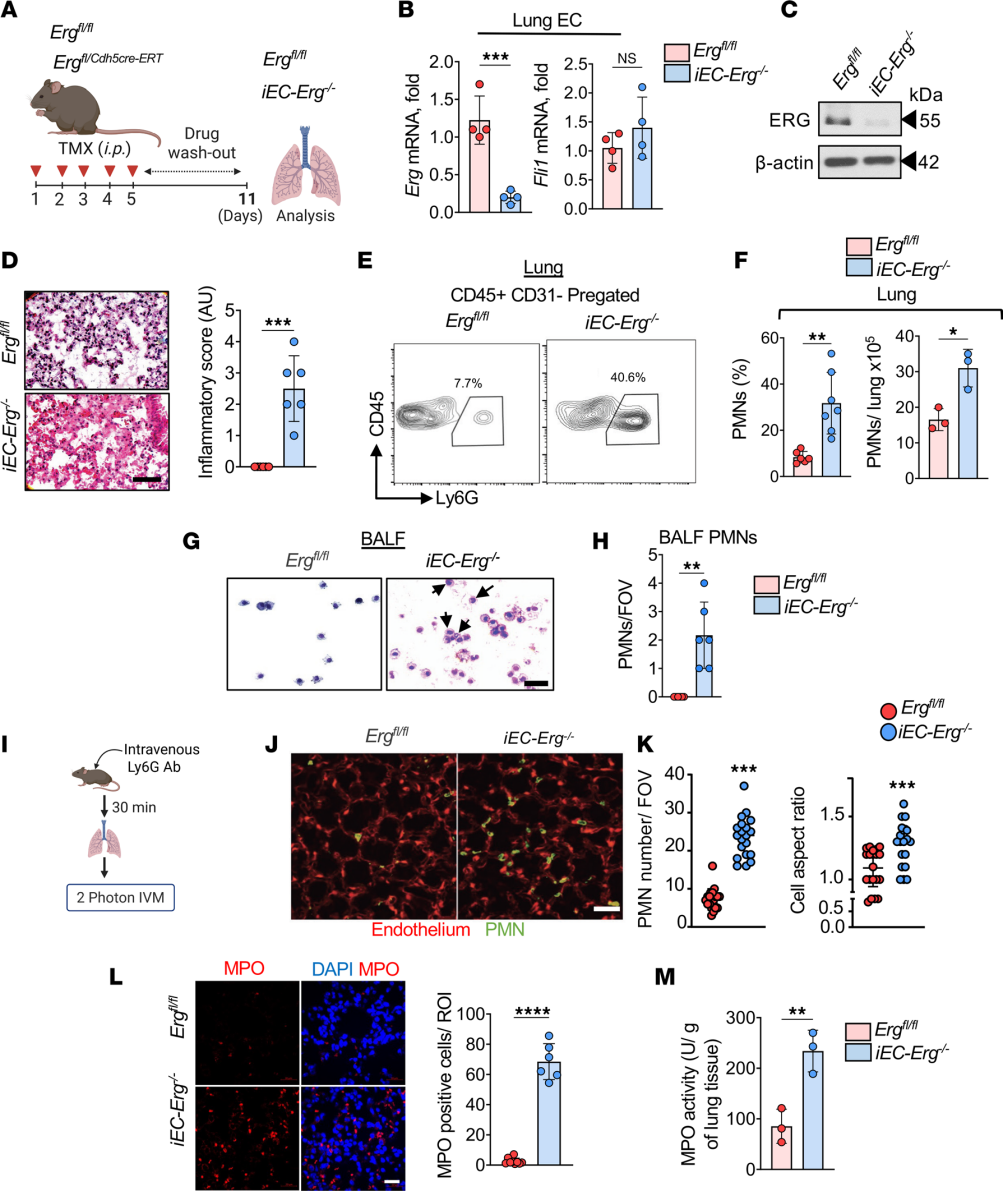
EC *Erg* loss induces PMN infiltration and activation. (**A**) Schematics of tamoxifen injection and drug washout as described in the Methods in 5-week-old mice. (**B** and **C**) mRNA levels of indicated genes in ECs sorted from the lungs (**B**) and ERG protein (**C**) of indicated mice (*n* = 6). *Gapdh* was used as an internal control in **B**, while actin was used as a loading control for protein in **C**. Numbers indicate densitometric analysis. (**D**) Lung histopathology and inflammatory score of *Erg^fl/fl^* and *iEC-Erg^–/–^* lungs (*n* = 6). Scale bar: 20 μm. (**E** and **F**) Flow cytometric analysis of PMNs in the indicated lungs. (**E**) Representative FACS plot. (**F**) PMN percentage (*n* = 8) and absolute count (*n* = 3). (**G** and **H**) H&E-stained BAL from indicated mice (*n* = 6). (**G**) Representative images. (**H**) Quantification of PMNs per field of view (FOV). Scale bar: 100 μm. (**I**–**K**) Schematics (**I**) and intravital 2-photon analysis of PMNs (green) and ECs (red) in the indicated lungs following i.v. injection of BV421-labeled anti-Ly6G antibodies (for PMNs) (**J** and **K**). Red ECs were confirmed using SeTau647-labeled anti-CD31 antibodies (not shown). The lungs were imaged 30 minutes after the administration of the antibody. Scale bar: 50 μm. (**J**) Representative images. (**K**) PMN cell number and aspect ratio (FOV = 298 × 298 mm) (*n* = 6). Experiments were performed twice independently. (**L** and **M**) MPO immunostaining of *Erg^fl/fl^* and *iEC-Erg^–/–^* lungs (*n* = 3). The plot shows MPO^+^ cells, which represent mean MPO fluorescence intensity/region of interest, pooled together from 3 lungs/group performed independently. (**L**) Representative images (MPO, red; nuclei, blue [DAPI]) and quantitation of MPO^+^ cells. Scale bar: 10 μm. (**M**) MPO activity of *Erg^fl/fl^* and *iEC-Erg^–/–^* lung tissues. Data represented as mean ± SD. *P < 0.05, **P < 0.01, ***P < 0.001, ****P < 0.0001 by unpaired, 2-tailed Student’s *t* test (all plots). NS, not significant.

ECs are ideally positioned to instruct immune cell infiltration and activation in the tissues (32). Because the lung, being a highly vascularized organ, is susceptible to infection at each breath, and neutrophilic inflammation causes lethal lung injury (2, 4, 14), we focused our studies on the lung as a model to determine the role of *Erg* in ECs in regulating immune cell functions. Using publicly available data, we confirmed that ECs predominantly express *Erg* in the lungs compared with other cell types (Supplemental Figure 1A; supplemental material available online with this article; https://doi.org/10.1172/jci.insight.195989DS1). Conditional deletion of *Erg* in ECs of the lungs of adult *Erg^fl/Cdh5-cre-ERT^* mice (Figure 1A) markedly decreased *Erg* mRNA levels in sorted ECs without altering the expression of a closely related ETS transcription factor, *Fli1* (Figure 1B). Immunoblotting and confocal imaging confirmed the deletion of ERG in pulmonary vessels (Figure 1C and Supplemental Figure 1B). In agreement with previous studies (25), lungs from tamoxifen-inducible EC-specific *Erg*-null (*iEC-Erg^–/–^*) mice were hemorrhagic, edematous, and inflamed regardless of sex (Figure 1D and Supplemental Figure 1C).

To investigate the impact of *Erg* deletion in ECs on the number of immune cells in the lungs, we performed flow cytometric analyses of ECs (CD31^+^CD45^–^) and hematopoietic cells (CD31^–^CD45^+^) in *Erg^fl/fl^* and *iEC-Erg^–/–^* lungs (Supplemental Figure 2A). Compared with *Erg^fl/fl^* lungs, *iEC-Erg^–/–^* lungs showed increased cellularity with reduced EC number, while hematopoietic cells were increased (Supplemental Figure 2, B and C). Further analysis of hematopoietic cells using established markers revealed an approximately 7-fold increase in PMN accumulation in the *iEC-Erg^–/–^* lungs but not in macrophages, platelets, or lymphocytes (Figure 1, E and F, and Supplemental Figure 2D). The frequency of PMNs was also increased in bone marrow and blood (Supplemental Figure 2E).

Bronchoalveolar lavage (BAL) showed that accumulated PMNs infiltrated the airspace (Figure 1, G and H). Additionally, we performed intravital 2-photon imaging of PMNs in the lungs in situ using anti-Ly6G antibodies in the mice with red *iEC-Erg^–/–^* ECs (using R26R*^tdTomato^Erg-cdh5^CreERT2^* mice in which tamoxifen injection turns on tomato expression in ECs lacking *Erg*) (Figure 1I). We found a significantly increased number of PMNs in the lungs of *iEC-Erg^–/–^* mice, consistent with our flow cytometric analyses (Figure 1, I–K). As anticipated, PMNs quickly circulated throughout the blood vessels in the control lungs, but these PMNs spread out and adhered to the blood vessels in the lungs lacking *iEC-Erg* (Figure 1K). *iEC-Erg^–/–^* PMNs also showed markedly elevated myeloperoxidase (MPO) activity and ROS levels compared with controls (Figure 1, L and M, and Supplemental Figure 2F). Together, these results suggest that conditional *Erg* deletion in ECs increased the accumulation of activated PMNs in the extravascular space and inflammatory vascular injury.

### Erg deletion in ECs alters the PMN transcriptome enriched with pathways inducing tissue recruitment, adhesion, and activation.

**Figure 2:**
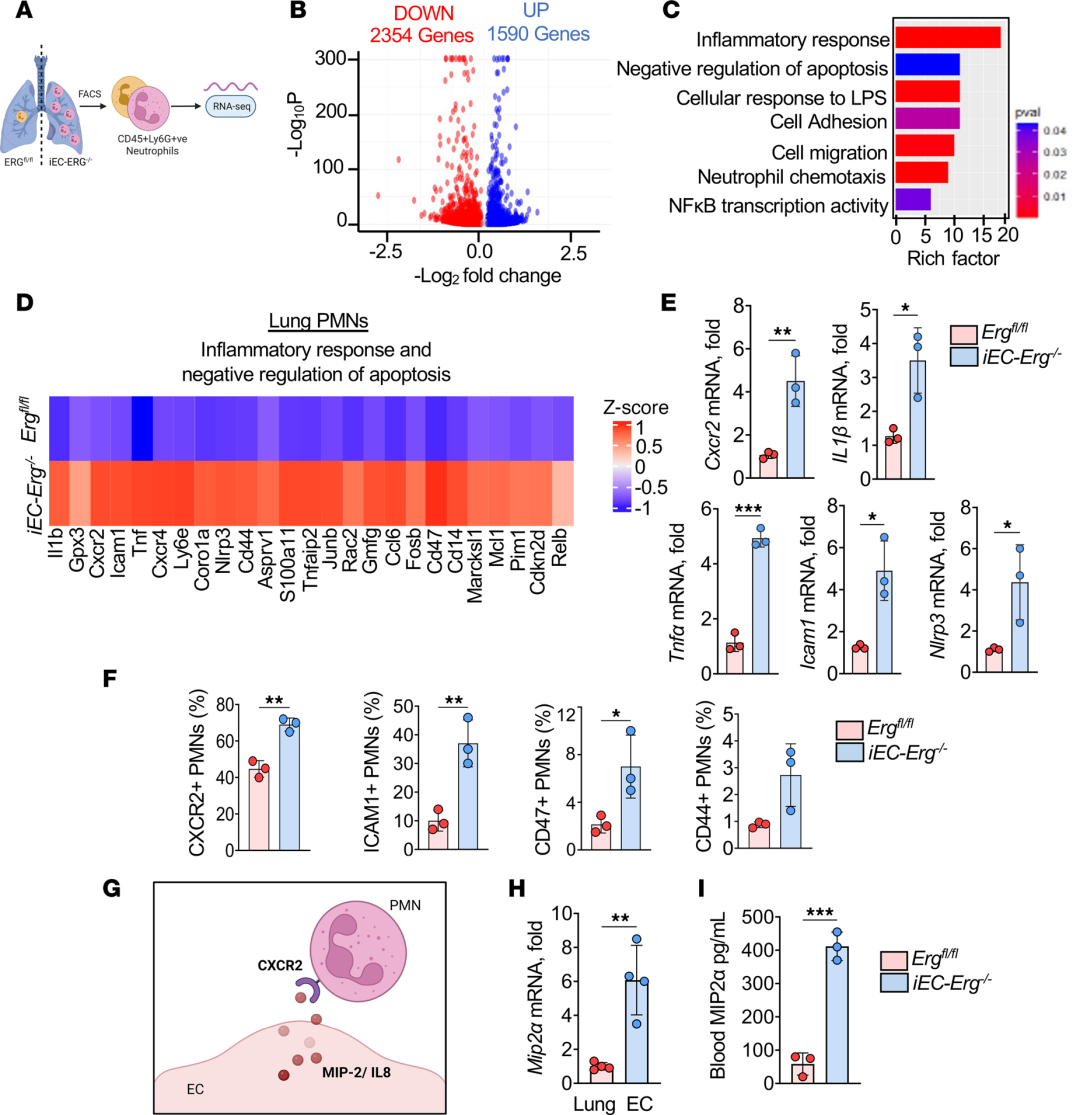
PMN transcriptome is shaped by the loss of *Erg* in ECs. (**A**) Schematics of RNA-seq from PMNs sorted from the lungs of 6 mice per group (pooled together). (**B**–**D**) Volcano plot (**B**), out of which the top 500 genes were selected for pathway enrichment analysis (**C**). (**D**) Heatmap of 25 upregulated genes from pathways indicated in **C**. (**E**) Validation of PMN mRNA expression using *Gapdh* as an internal control (*n* = 3). (**F**) Cell surface expression of indicated genes in PMNs using flow cytometry (*n* = 3). (**G**–**I**) Schematics (**G**), *Mip2a* mRNA in ECs sorted from the lungs (**H**), and blood *Mip2a* levels (**I**) in indicated mice (*n* = 3). *Gapdh* was used as an internal control for mRNA. Data represented as mean ± SD. *P < 0.05, **P < 0.01, ***P < 0.001 by unpaired, 2-tailed Student’s *t* test (all plots).

The PMN transcriptome changes during circadian variation, cancer metastasis, cardiovascular diseases, and autoimmune diseases (7, 8, 33–35). We next explored whether the infiltrated PMN transcriptome was altered in *iEC-Erg^–/–^* lungs (Figure 2A). A heatmap and volcano plot from RNA sequencing (RNA-seq) revealed that, out of 3945 differentially expressed genes, 1590 genes were upregulated in PMNs from *iEC-Erg^–/–^* lungs compared with control lungs (Figure 2B and Supplemental Figure 3A). Pathway analysis of the top 500 transcripts revealed that *iEC-Erg^–/–^* PMNs are enriched with genes associated with pathways that activate inflammatory responses, migration, chemotaxis, and the negative regulation of apoptosis, corroborating the above functional findings (Figure 2C). Because PMNs from *iEC-Erg^–/–^* lungs were highly adhesive and activated, we assessed the top upregulated genes from the above-mentioned pathways, as shown in Figure 2D. These genes included *Cxcr2*, *Il1b*, *Tnfa, Icam1*, and *Nlrp3*, which we validated using qPCR in PMNs sorted from control and *iEC-Erg^–/–^* lungs (Figure 2E). CXCR2 is known to be critical for recruiting PMNs to the site of injury, while ICAM1 and CD44 are crucial for regulating leukocyte extravasation and adhesion to endothelium (4, 36, 37). CD47 expression in PMNs is a “do not eat me” signal, which delays PMN apoptosis and phagocytic clearance (38). Indeed, we found increased frequency of CXCR2-expressing PMNs in the blood of *iEC-Erg^–/–^* mice (Supplemental Figure 3B). FACS analysis also showed increased cell surface expression of CXCR2, ICAM1, CD44, and CD47 on PMNs in *iEC-Erg^–/–^* lungs (Figure 2F and Supplemental Figure 3, C–E). Thus, the loss of EC *Erg* was associated with the presence of inflammatory, adhesive, and long-lived PMNs in the lungs due to the upregulation of several genes, including *Cxcr2*, *Icam1*, *Cd44*, and *Cd47*.

MIP2α/IL-8 is the predominant chemokine activating CXCR2 on PMNs (39). Evidence also indicates that ERG represses the transcription of IL-8 in human umbilical vein–derived ECs (HUVECs) (18). We therefore assessed MIP2α levels in ECs sorted from the lungs of mice lacking *Erg* (Figure 2G). We found that the *Mip2a* transcript was increased by 6- to 7-fold in male or female *iEC-Erg^–/–^* lungs or ECs sorted from the lungs of male or female *iEC-Erg^–/–^* mice (Figure 2H and Supplemental Figure 3F). Further experiments demonstrated that ECs secreted MIP2α, as indicated by elevated levels of this cytokine in the blood of *iEC-Erg^–/–^* mice at homeostasis (Figure 2I).

These results prompted us to investigate the time course of *Erg* deletion in the ECs of adult mice and the resulting change in PMN numbers in the lungs (Supplemental Figure 4A). Tamoxifen administration induced a significant decrease in *Erg* mRNA in the lungs even on the third day (Supplemental Figure 4B). These differences translated into increased lung inflammation, as reflected by increased *Mip2a* mRNA expression in *iEC-Erg^–/–^* lungs (Supplemental Figure 4C), which was then associated with an increase in PMNs (Supplemental Figure 4, D and E).

### PMNs sorted from iEC-Erg^–/–^ lungs retain inflammatory memory in naive recipients.

**Figure 3:**
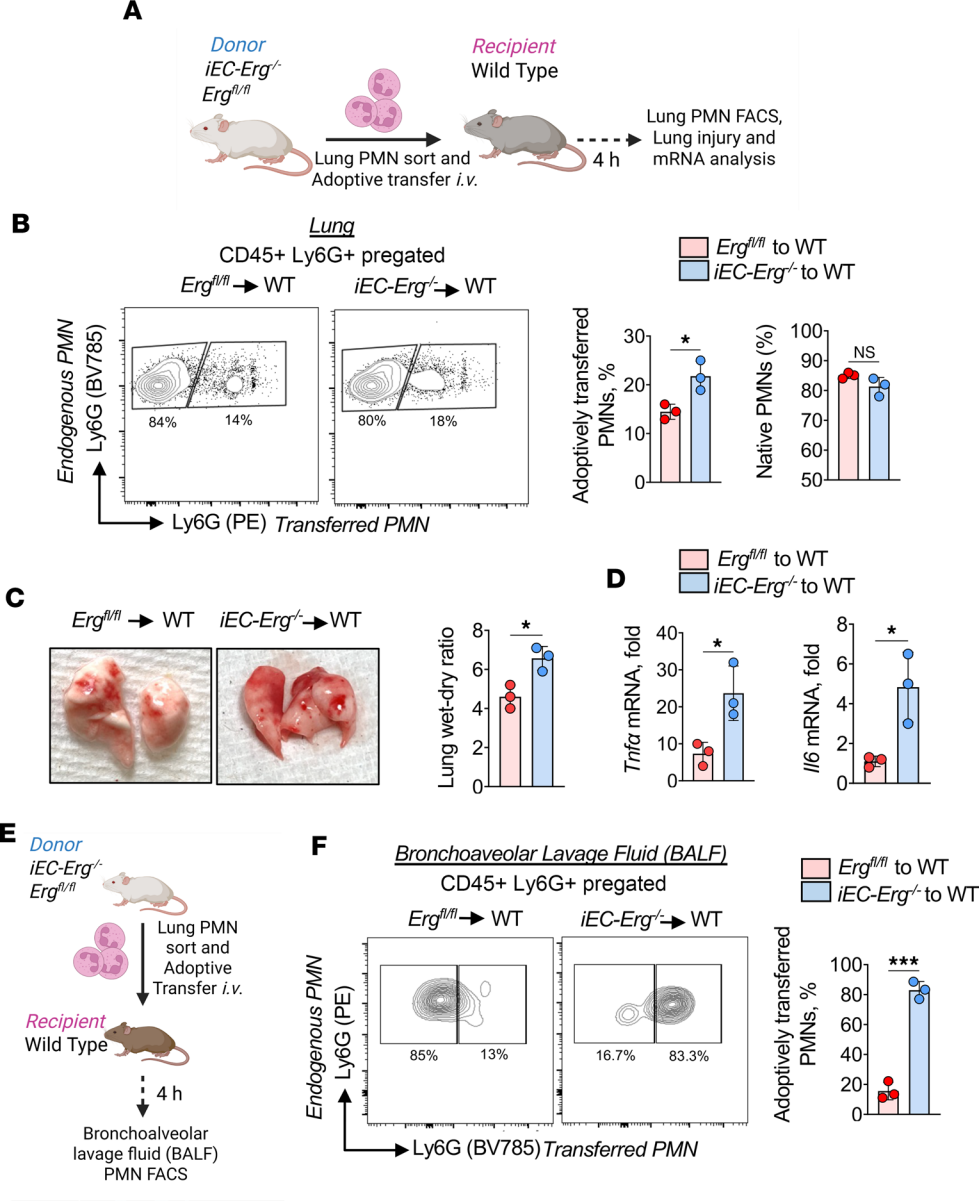
Adoptively transferred PMNs from *iEC-Erg^–/–^* mice induce lung injury. (**A**) Schematic diagram showing flow sorting of lung PMNs from control and *iEC-Erg^–/–^* mice followed by adoptive transfer of labeled PMNs (i.v.) in naive WT mice for lung PMN analysis and vascular injury as well as cytokine expression. (**B**) FACS analysis of WT mouse lung shows the increased percentage of adoptively transferred PMNs in the lungs (*n* = 3). (**C**) Lung wet-dry ratio in the recipient mice. Left shows a representative image, while right shows the quantitation. (**D**) mRNA expression of indicated genes in the recipient’s lungs using *Gapdh* as an internal control (*n* = 3). (**E**) Protocol of assessment of extravasation of *iEC-Erg^–/–^* PMNs in the airspace of mouse lungs. PMNs sorted from control and *iEC-Erg^–/–^* mice were adoptively transferred i.v. into WT mice, after which BALF PMNs were analyzed. (**F**) FACS plots (left), while the right shows quantitation (*n* = 3). Data represented as mean ± SD. *P < 0.05, ***P < 0.001 by unpaired, 2-tailed Student’s *t* test (all plots).

Activated PMNs disrupt vascular homeostasis (40). Based on our finding that deletion of *Erg* in ECs shifted the PMN transcriptome toward an inflammatory lineage, we next tested whether these PMNs would induce lung vascular inflammatory injury following their adoptive transfer in naive mice. We immunostained the lung cell suspension from control and *iEC-Erg^–/–^* mice using a phycoerythrin-conjugated (PE-conjugated) anti-Ly6G antibody (Figure 3A). After sorting, the cells were adoptively transferred (1 × 10^6^ i.v.) into naive wild-type (WT) mice. We determined lung and BAL fluid (BALF) PMN frequencies, endothelial injury, and inflammatory gene expression 4 hours after transplantation. Notably, the frequency of donor *iEC-Erg^–/–^* PMNs was approximately 1.7-fold higher (a 55% difference) than that of control donor PMNs in the lungs of naive recipient mice (Figure 3B). In contrast, the frequency of pulmonary naive PMNs in the recipient mice was unaffected (Figure 3B). Notably, transplanted *iEC-Erg^–/–^* PMNs led to inflammatory lung injury in naive recipient mice, as indicated by an increase in lung edema and expression of inflammatory cytokines (Figure 3, C and D). In other studies, we also assessed whether PMNs transferred from *iEC-Erg^–/–^* mice to naive mice extravasated into the airspace (Figure 3E), as observed in the *iEC-Erg^–/–^* mouse in Figure 1, G and H. Indeed, we found an approximately 5-fold increase in accumulation of adoptively transferred PMNs from *iEC-Erg^–/–^* mice in the airspace compared with control donors (Figure 3, E and F). These findings further support the notion that PMNs from *iEC-Erg^–/–^* mice are highly inflammatory and induce lung vascular barrier disruption.

### Inhibiting PMN CXCR2 activity reduces inflammatory injury in iEC-Erg^–/–^ mice.

Reparixin is a high-affinity allosteric inhibitor of CXCR1 and CXCR2 (41). To assess whether CXCR2 regulated PMN activity and function in *iEC-Erg^–/–^* mice, we first used *Erg*-depleted ECs and cocultured them with PMNs derived from HL-60 cells without or with reparixin treatment (Supplemental Figure 5A). Consistently, PMNs showed increased *Cxcr2* mRNA, which was reduced upon reparixin treatment (Supplemental Figure 5B). As in lungs (Figure 1, I and J), approximately 5-fold higher PMN attachment was found in *Erg*-depleted ECs than in control ECs (Supplemental Figure 5C), which was reduced by reparixin (Supplemental Figure 5C).

**Figure 4:**
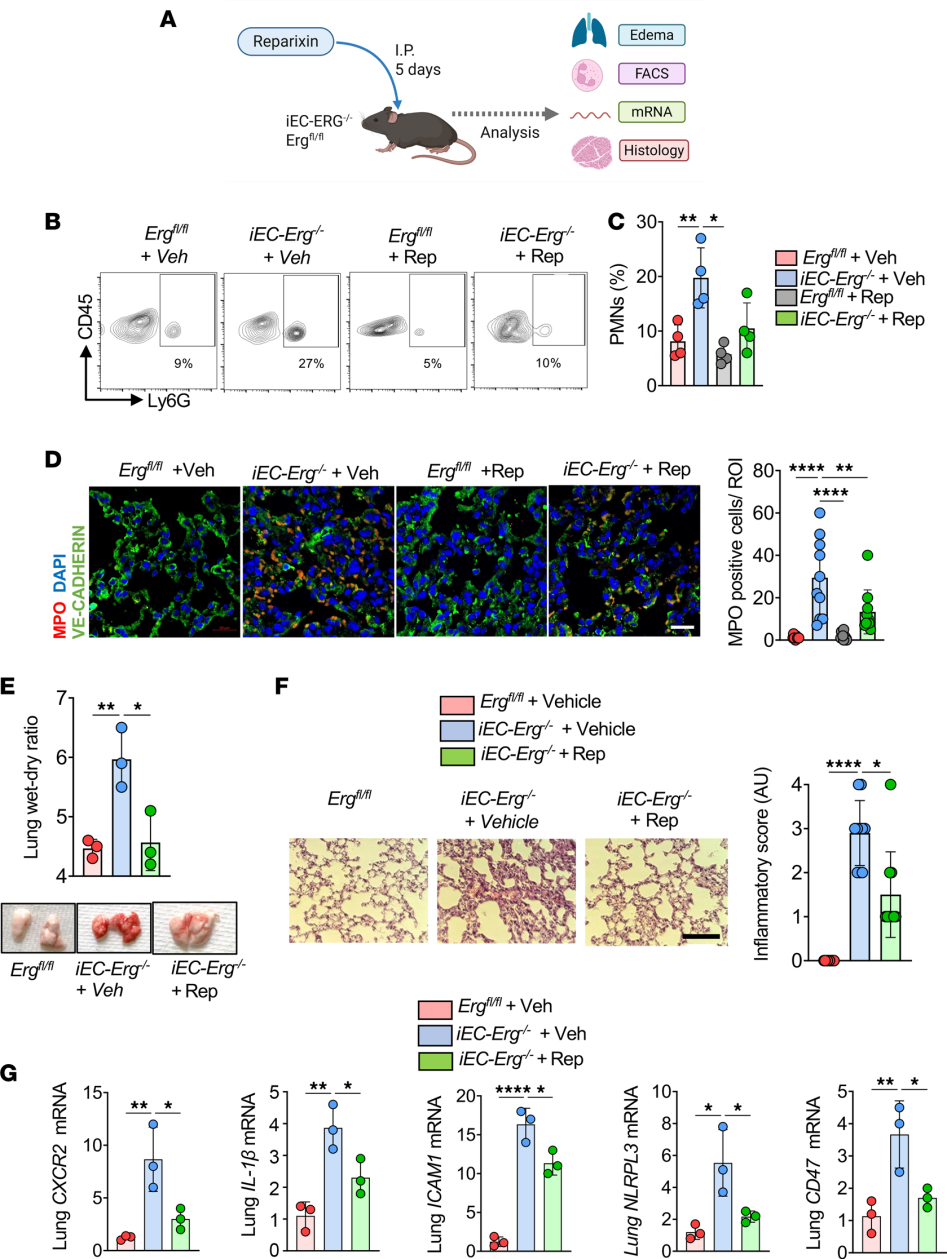
CXCR2 antagonism resolves neutrophilic lung injury in *iEC-Erg^–/–^* mice. (**A**) Experimental design showing treatment of *iEC-Erg^–/–^* mice with reparixin (i.p.) for 5 days daily. (**B** and **C**) PMN analysis showing representative FACS plots (**B**) and their quantification (**C**) (*n* = 4). (**D**) MPO immunostaining. The left shows representative images (MPO, red; VE-cadherin, green; nuclei, blue, DAPI), while the right shows quantification. Scale bar: 20 μm (*n* = 6). (**E**) Lung wet-dry ratio. The top shows the quantitation, while the bottom shows hemorrhagic lungs (*n* = 3). (**F**) Lung H&E staining (left) and the corresponding inflammatory score are shown on the right (*n* = 6). (**G**) Inflammatory cytokine expression in lungs. Data represented as mean ± SD. *P < 0.05, **P < 0.01, ****P < 0.0001 by 1-way ANOVA, followed by Tukey’s multiple-comparison test (all plots).

Having identified that reparixin blocked PMN adhesion and accumulation in the lungs, we next injected reparixin along with tamoxifen in *Erg^cdh5Cre-ERT2^* mice, and subsequently determined PMN numbers and lung inflammation (Figure 4A). Notably, reparixin reduced PMN numbers and lung MPO activity (Figure 4, B–D) and decreased lung edema and inflammatory score in *iEC-Erg^–/–^* lungs toward control levels (Figure 4, E and F). Reparixin also reduced the expression of several cytokines in the *iEC-Erg^–/–^* lungs, which we had previously shown to be upregulated in the PMNs, including CXCR2 and ICAM1 (Figure 4G). These observations suggest that the loss of *Erg* in ECs, which involves the generation of IL-8/MIP2α and the activation of PMN CXCR2, is a crucial mechanism underlying lung damage in *iEC-Erg^–/–^* mice.

### EC ERG controls neutrophilic lung injury by transcribing A20.

**Figure 5:**
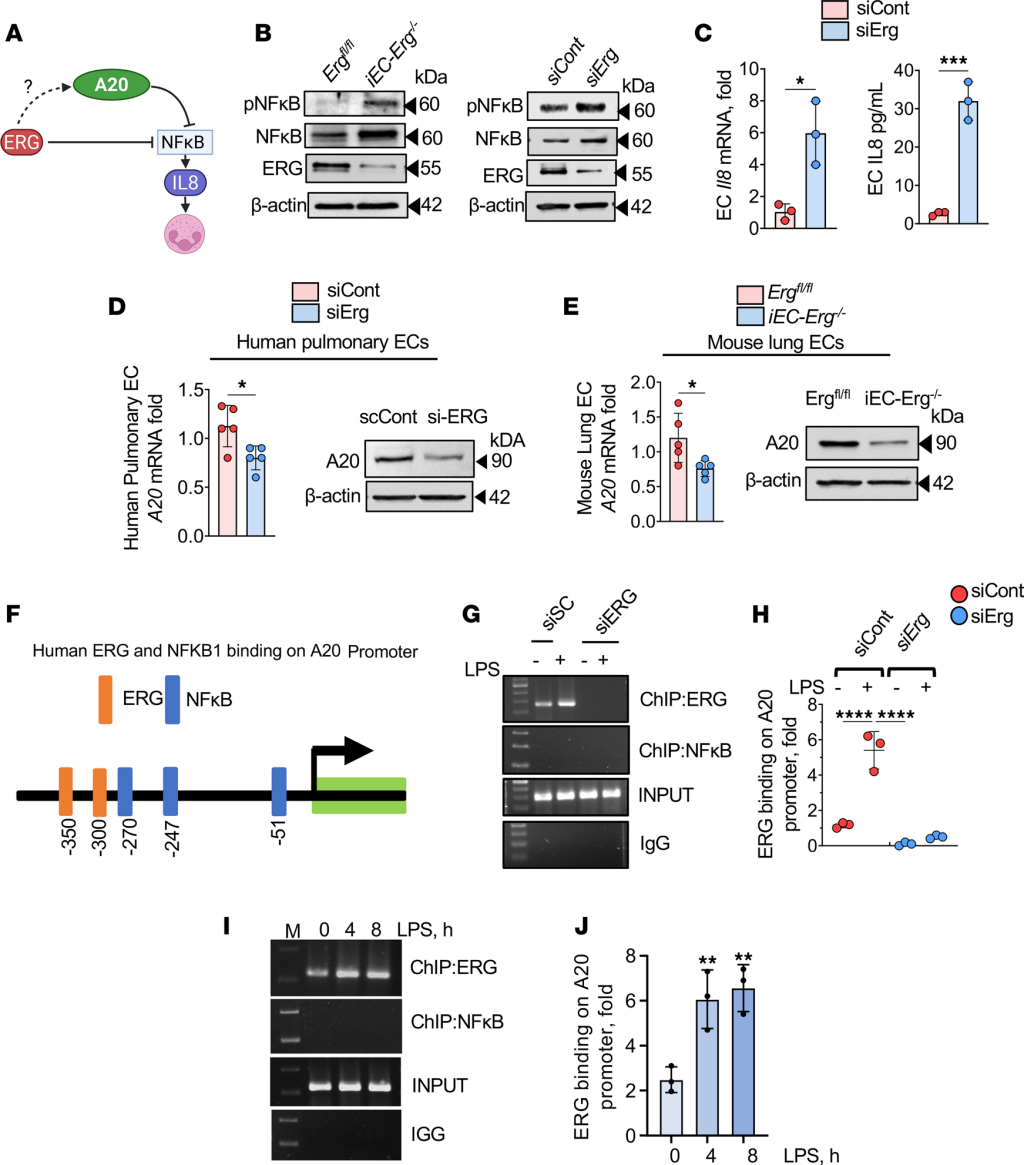
*ERG* controls IL-8 secretion from ECs and PMN infiltration by upregulating A20 expression. (**A**) Model of *ERG*/A20 regulation of PMN activation. (**B**) Immunoblots showing NF-κB expression and phosphorylation in *Erg*-null lungs (left) and *ERG*-depleted ECs (right). Actin was used as a loading control. (**C**) *Mip2a* mRNA (left) and protein levels (right) from control or *Erg*-depleted ECs. *Gapdh* was used as an internal control. (**D** and **E**) A20 mRNA (left) and protein (right) expression in the indicated ECs (**D**) and lungs (**E**). *Gapdh* was used as an internal control for mRNA (*n* = 5), while actin was used as a loading control for protein. A representative immunoblot is shown from experiments that were repeated 3 times. (**F**) In silico analysis of A20 promoter showing *ERG* and NF-κB binding sites. (**G**–**I**) ChIP analysis of A20 with *ERG* and NF-κB in *ERG*-depleted (**G** and **H**) or LPS-stimulated (**I** and **J**) ECs. **G** and **I** show representative blots, while **H** and **J** show the quantitation of PCR products. Data represented as mean ± SD. *P < 0.05; **P < 0.01; ***P < 0.001; ****P < 0.0001 by unpaired, 2-tailed Student’s *t* test (**C**–**E**) and 1-way ANOVA followed by Tukey’s multiple-comparison test (**H** and **J**).

We next sought to identify the molecular basis of *Erg* suppression of MIP2α/IL-8 secretion from ECs. NF-κB, a crucial transcription factor that induces inflammatory signaling downstream of diverse pathogens in ECs, synthesizes IL-8/MIP2α during injury, in part by competing with ERG for binding to the *IL8* promoter (18, 20). Therefore, we addressed the possible role of *Erg* in suppressing NF-κB activity and, thereby, PMNs’ activation (Figure 5A). We found that *iEC-Erg^–/–^* lungs spontaneously increased NF-κB protein and phosphorylation levels, a measure of NF-κB activity (42) (Figure 5B and Supplemental Figure 6A). *ERG* depletion in human ECs similarly induced NF-κB signaling (Figure 5B and Supplemental Figure 6B). Moreover, we found that *ERG* depletion increased basal levels of IL-8 mRNA and protein (Figure 5C). These findings suggest that *ERG* represses basal NF-κB activity to prevent the generation of IL-8/MIP2α and maintains an anti-adhesive vascular niche, thereby suppressing PMN accumulation and activation.

Deubiquitinases are essential in interfacing with and modifying ubiquitinated proteins to curb persistent NF-κB activation (43, 44). Because *Erg* deletion in ECs spontaneously induced NF-κB activity, we tested the hypothesis that *ERG* regulates EC NF-κB activity and, thereby, IL-8/MIP2α secretion by modulating deubiquitinases. We screened deubiquitinases, including CYLD, USP9X, USP18, and A20 (also known as TNFAIP3) (44) in ECs, and found that *ERG* depletion in human lung ECs reduced A20 mRNA levels by approximately 30% without altering the expression of other deubiquitinases (Figure 5D and Supplemental Figure 6C). Depletion of *ERG* reduced A20 protein levels by 80% (Figure 5D and Supplemental Figure 6D, left). ECs sorted from control and *iEC-Erg^–/–^* lungs similarly showed significantly reduced A20 mRNA expression and protein levels (Figure 5E and Supplemental Figure 6D, right).

LPS increases A20 expression, and the loss of A20 in ECs induces vascular inflammatory injury (45). NF-κB is known to induce A20 synthesis during inflammation to negatively control its excessive signaling (46–48). In silico analysis showed that within 1 kb of the A20 promoter, *ERG* has 2 binding sites (–350 and –300 nt). In contrast, NF-κB has 3 binding sites (–270, –247, and –51 nt) (Figure 5F). We therefore performed sequential chromatin immunoprecipitation (SeqChIP) analysis to assess the binding of *ERG* versus NF-κB on the A20 promoter using ECs stimulated with LPS or *ERG*-depleted ECs. We found that *ERG* bound A20 basally; however, this response was not observed in *ERG*-depleted ECs (Figure 5, G and H). LPS increased *ERG* binding on the A20 promoter (Figure 5, I and J). However, we failed to find NF-κB binding on the A20 promoter in *ERG*-depleted ECs or ECs stimulated with LPS (Figure 5, G–J).

**Figure 6:**
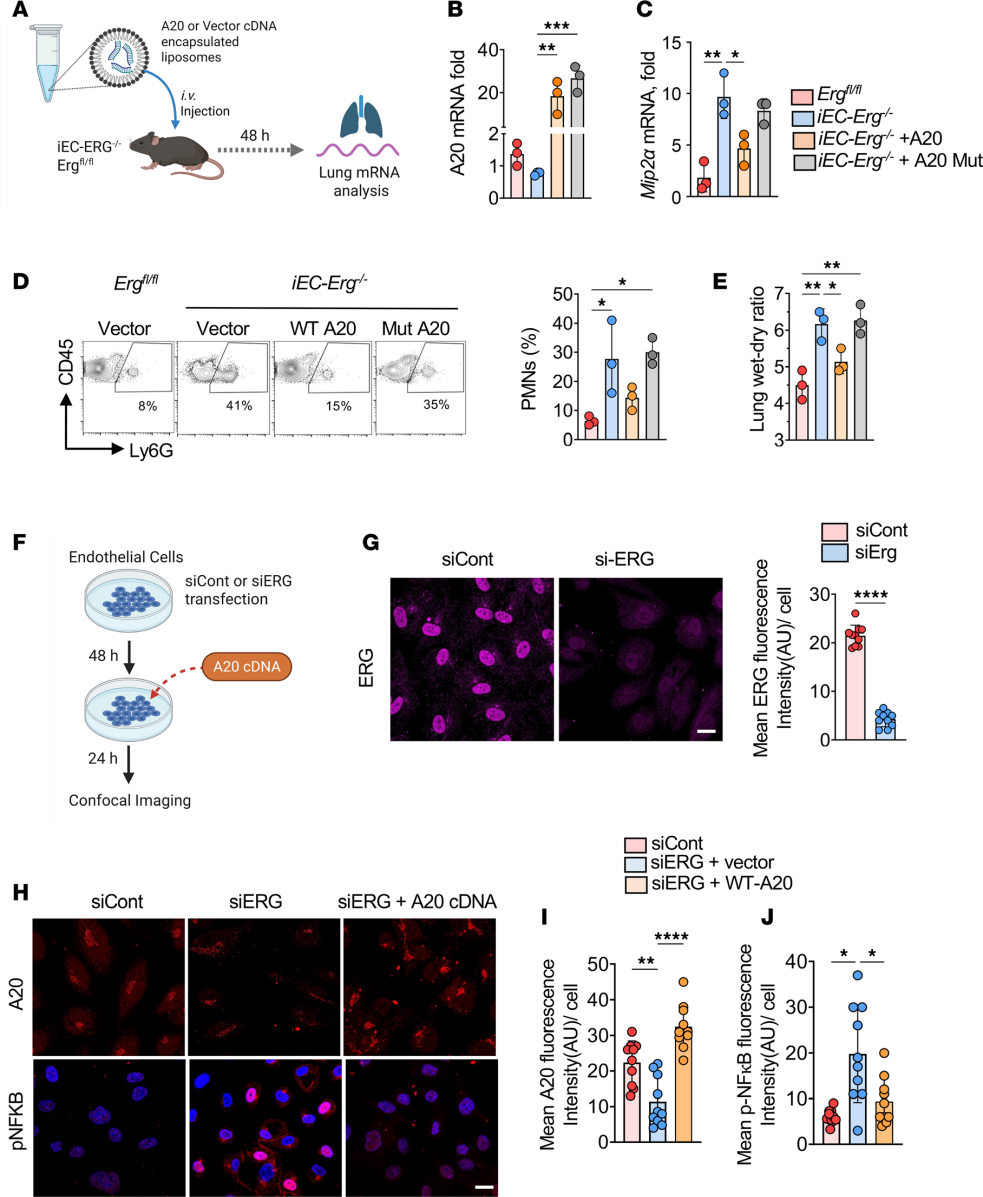
A20 overexpression in *Erg*-null endothelium suppresses PMN infiltration and resolves inflammatory lung injury. (**A**) Schematics of gene delivery using liposomes in *iEC-Erg^–/–^* mice on day 11 and assessment of injury as indicated. (**B**) Lung A20 mRNA expression using *Gapdh* as an internal control. *n* = 3 lungs/group. (**C**) Lung *Mip2a* mRNA, using *Gapdh* as the loading control. (**D**) PMN analysis showing FACS plots (left) and quantitation (right), where *n* = 3. (**E**) Lung edema was determined by quantifying the lung wet-dry ratio. Data represented as mean ± SD. (**F**) Experimental plan. (**G**) *Erg* expression in indicated ECs. The left image shows a representative example, while the right image displays the quantification. Scale bar: 20 μm. (**H** and **I**) Representative images (**H**) and quantitation (**I**) of A20 and NF-κB activity in indicated ECs. Scale bar: 20 μm. *P < 0.05; **P < 0.01; ***P < 0.001; ****P < 0.0001 by unpaired, 2-tailed Student’s *t* test (**G**) and 1-way ANOVA followed by Tukey’s multiple-comparison test (**B**, **C**, **E**, **I**, and **J**).

To assess the causal role of A20 deubiquitinase in reversing the EC inflammatory phenotype, we determined whether restoring A20 expression in ECs of *iEC-Erg^–/–^* mice would bypass the *ERG* requirement, thus reducing MIP2α/IL-8 levels, neutrophilic inflammation, and resolving vascular injury in *iEC-Erg^–/–^* mice. We first assessed the effect of overexpressing A20 in *Erg*-depleted ECs by transducing WT A20 or A20 cDNA lacking deubiquitinase activity (A20-DUB mutant) (Supplemental Figure 6E). We found that rescuing A20 expression reversed IL-8 mRNA and protein levels in *Erg*-depleted ECs to those seen in control cells. Still, this effect was not seen in the ECs transduced with the A20-DUB mutant (Supplemental Figure 6, F–H). Next, we complexed WT A20 or A20-DUB mutant cDNA driven by the *Cdh5* (VE-cadherin) promoter in liposomes (43, 49) to express *A20* only in ECs of *iEC-Erg^–/–^* mice (Figure 6, A and B) and quantified PMN number, *Mip2a* expression, and lung edema. Transducing WT A20 but not the A20-DUB mutant in ECs of *iEC-Erg^–/–^* lungs suppressed *Mip2a* (Figure 6C) and PMN accumulation (Figure 6D). Moreover, A20, but not the mutant, promoted the resolution of lung edema (Figure 6E). We also performed confocal analysis to investigate the impact of ERG loss in ECs on A20 expression and NF-κB activity (Figure 6, F and G). Confocal imaging showed that *Erg* depletion reduced A20 expression but increased accumulation of p-NF-κB in the nucleus (Figure 6, H and I). Intriguingly, overexpression of A20 in *Erg*-depleted ECs markedly decreased NF-κB activity (Figure 6, H–J). These findings corroborate the notion that *ERG*-mediated synthesis of A20 is required to suppress NF-κB activity, IL-8 generation, and PMN sequestration and activation.

### Therapeutically targeting CXCR2 activity suppresses neutrophilic lung injury and enhances survival in mice after bacterial pneumonia.

Increased levels of IL-8 and CXCR2 are associated with lung injury. In that case, patients with lung injury and mouse models of lung injury should display a reduction in EC *Erg* in the lungs and activation of the IL-8/CXCR2 cascade, which could be reversed by CXCR2 blockade or through A20 expression in ECs. Interestingly, we found a significant reduction in *ERG* expression in the pulmonary vessels of patients who died from lung injury (ARDS) compared with non-ARDS patients (Supplemental Figure 7A). In contrast, PMN activation, as reflected by increased MPO activity, was increased in the lungs of patients with ARDS (Supplemental Figure 7B). Next, we evaluated whether decreased *ERG* expression in the lungs of ARDS patients also altered gene expression in blood PMNs of ARDS patients in the ICU compared with control individuals. Patients with ARDS exhibited augmented mRNA expression of *Il6*, *Cxcr2*, and *Nlrp3* in blood PMNs compared with non-ARDS (Supplemental Figure 7C). Reanalysis of publicly available data similarly showed hyperactivated PMNs in lungs of patients suffering from severe COVID-19, as revealed by increased expression of several inflammatory genes, including CXCR2 (Supplemental Figure 7D).

**Figure 7:**
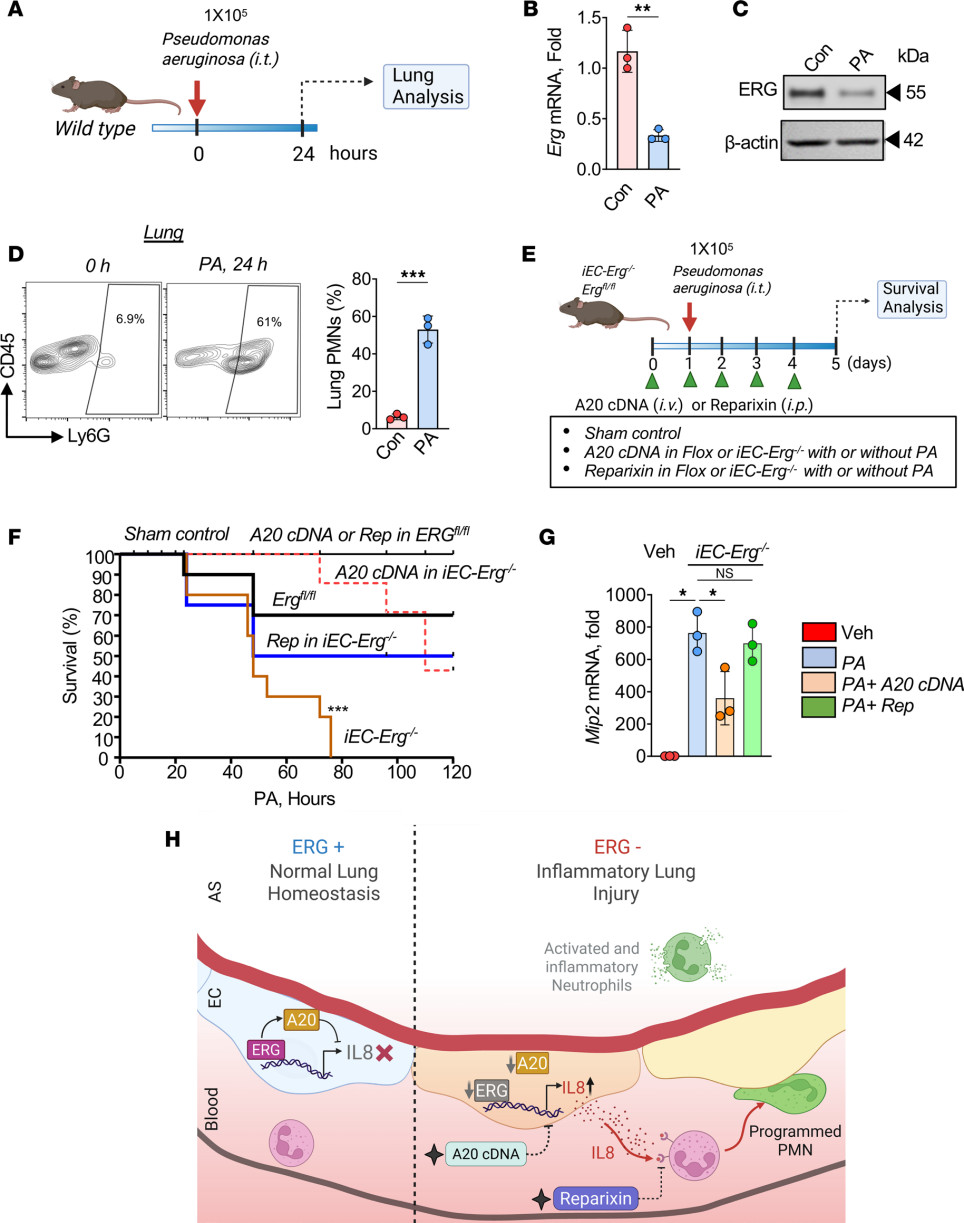
Bacterial pneumonia decreases endothelial *Erg* and promotes neutrophilic lung injury. (**A**) Experimental plan. *Erg* mRNA (**B**) and protein levels (**C**) at indicated times after *PA* challenge (*n* = 3). *Gapdh* was used as a control for mRNA, while actin was used as a loading control for protein. (**D**) PMN numbers in the *PA*-infected lungs at the indicated times. The left shows the FACS plot, while the right shows quantitation (*n* = 3). (**E**) Schematics. (**F**) Kaplan-Meier survival analysis in *PA*-infected animals. Mice received 1 × 10^5^ CFU of *PA*. Mouse survival was assessed every 6–12 hours after *PA* instillation, without or with therapy (*n* = 10). (**G**) *Mip2a* mRNA normalized to *Gapdh* (*n* = 3). (**H**) Proposed model showing the functional role of *ERG* in controlling IL-8 levels in ECs by transcribing A20 and suppression of CXCR2-mediated PMN activation and lung injury. A20 gene in ECs or blockade of CXCR2 activity using reparixin suppresses neutrophilic lung injury and enhances mouse survival after bacterial pneumonia. Data represented as mean ± SD. *P < 0.05; **P < 0.01; ***P < 0.001 by 1-way ANOVA followed by Tukey’s multiple-comparison test (all plots).

Pneumonia caused by *Pseudomonas aeruginosa* (*PA*) in hospitalized patients causes ALI, compromising their survival (50). We, therefore, used a well-characterized mouse model of lung injury where *PA* induces severe ALI within 12–24 hours (51) (Figure 7A), to assess its impact on *Erg* expression in association with PMN accumulation. *PA* infection reduced *Erg* expression levels markedly at the mRNA and protein levels within 24 hours after infection in the lung (Figure 7, B and C). This was associated with significant infiltration of PMNs in the lungs at 24 hours (Figure 7D).

Next, we investigated whether *PA* would compromise the survival of control and *iEC-Erg^–/–^* mice after *PA* infection, and whether blocking CXCR2 activity or delivering the A20 gene to the ECs of these mice would improve their survival (Figure 7E). *iEC-Erg^–/–^* mice received reparixin or the A20 gene in ECs using liposomes, following which these mice were infected with *PA* (Figure 7E). *PA* infection resulted in 100% mortality within 75 hours in *iEC-Erg^–/–^* mice, whereas 30% of control mice remained viable for up to 5 days (Figure 7F). We observed that the time course of mouse lethality from pneumonia was markedly shifted to the right in mice receiving A20 cDNA in ECs of *iEC-Erg^–/–^* mice (Figure 7F). Only 10% of the mice died at approximately 70 hours, while 30% were dead at 100 hours, with 40% of these mice surviving at 120 hours. In the case of reparixin, the mice died at a faster rate, with 25% of the mice dead at approximately 20 hours, which increased to 50% at 50 hours, with 50% of these mice surviving until 120 hours (Figure 7F). Additionally, A20 overexpression also reduced *Mip2a* expression in *iEC-Erg^–/–^* lungs. Still, this response was not observed in reparixin-treated groups (Figure 7G). These results indicate that the A20 ERG–mediated decrease in IL-8/MIP2α generation in ECs is a key step in reducing neutrophilic inflammation and ALI lethality.

## Discussion

Here, we investigated the endothelial mechanisms that restrict PMN accumulation and activation in tissues, with the possibility of exploiting this mechanism for therapeutic applications during lung injury. Understanding the pathway that prevents hyperactivation of PMNs is essential for maintaining organ function, such as the lungs, which are continually exposed to environmental pathogens with each breath. Unwanted PMN activation can compromise fluid-tissue homeostasis.

Our study revealed that the basal activity of *ERG*, a transcription factor that has a well-described role in maintaining EC homeostasis during development and adulthood (15, 24, 28, 30, 52), controls PMN transcriptome for aberrant tissue accumulation and activation. We demonstrated that PMN infiltration at a steady state into the airspace correlated with decreased *Erg* expression in ECs, resulting in the synthesis and release of MIP2α/IL-8 into the blood. Moreover, a substantial loss of *Erg* during *PA* infection in mice and in lungs from patients with ARDS was also associated with neutrophilic injury. PMNs from *iEC-Erg^–/–^* lungs were hyperactive, characteristic of inflammatory injury (53). Our data indicate that vascular *Erg* could be a unique molecular mechanism that regulates the PMN transcriptome. PMNs isolated from *iEC-Erg^–/–^* lungs exhibited upregulation of genes associated with pathways that induce inflammation, chemotaxis, and cell survival. In this context, we found increased expression of genes, including *Cxcr2, Icam1, Cd44,* and *Cd47*, in *iEC-Erg^–/–^* PMNs recruited to the airspace compared with control PMNs. CXCR2 in PMNs regulates their chemotaxis (39), while CD44 prolongs PMN survival and disease severity (8). Crucially, these genes were also expressed in PMNs from patients with ARDS.

Interestingly, we also demonstrated that adoptively transferred *iEC-Erg^–/–^* PMNs induced inflammatory lung injury in naive mice without an insult, despite normal *Erg* levels. PMNs encounter pulmonary circulation as the initial vascular bed (54, 55) upon adoptive transfer, where they are mobilized and lodged in substantial numbers due to the narrow capillaries (56, 57). We speculate that the primed “inflammatory and adhesive” state of *iEC-Erg^–/–^* PMNs enables enhanced and prolonged interaction with pulmonary vessels, leading to their preferential retention in the lungs and contributing to inflammatory injury. Since cytokines such as TNF-α, IL-1β, and IL-6 can target *ERG* expression (25), we also speculate that these inflammatory PMNs decrease *ERG* levels in ECs. Loss of EC *Erg* leads to vascular hyperpermeability (27), which can induce PMN infiltration and activation (58). These combined mechanisms likely explain the observed effect of *iEC-Erg^–/–^* PMNs in normal lungs in the absence of exogenous inflammatory insults, but warrant further investigation.

Recent studies showed that PMNs squeeze across the vasculature, which converts their transcriptome, favoring phagocytic capacity (59). We showed that PMNs acquired an inflammatory lineage following the deletion of *Erg* in ECs within 3 days, and these mice subsequently died from pneumonia. However, inhibiting CXCR2 activity with reparixin, an allosteric inhibitor of CXCR2/CXCR1 (41), reduced PMN number in the lungs of *iEC-Erg^–/–^* mice and restored PMNs toward the antiinflammatory lineage, resulting in markedly improved lung fluid homeostasis in *iEC-Erg^–/–^* mice. In line with these findings, reparixin also suppressed PMN accumulation in the lungs of control mice and prevented their death after pneumonia. Reparixin also improved the survival of *iEC-Erg^–/–^* mice after pneumonia. Given our findings that reduced *ERG* and increased PMN activity were observed in patients dying of ARDS and in patients with severe SARS-CoV-2–induced lung injury, our results demonstrate that therapeutically targeting the CXCR2 cascade using reparixin during pneumonia can be exploited to improve patients’ mortality from ALI.

Several transcription factors, including NF-κB, can induce IL-8 expression during injury (20, 60). *ERG* was shown to bind the IL-8 promoter shared by NF-κB (19). However, NF-κB is not basally active, indicating other mechanisms must be involved to upregulate IL-8 synthesis by NF-κB upon the loss of *ERG*. We demonstrated reduced A20 expression in *Erg*-null ECs. A20 is a deubiquitinase that regulates multiple functions of ECs through suppressing NF-κB activity (43, 44, 49). Reduced A20 is known to be involved in diabetes, vasculitis, retinitis, and lung injury (43, 61–65). We showed that *ERG* binds to the A20 promoter. Rescuing the deubiquitinase expression and activity in *Erg^–/–^* ECs and lungs suppressed MIP2α/IL-8 secretion from ECs, decreased PMN accumulation and their activation in the lungs, reinforcing that the *ERG*-induced A20 synthesis was the yet-to-be-unidentified arm in *ERG*-dependent control of EC-PMN homeostasis. Thus, our results demonstrate that A20 can bypass *ERG* to reverse the EC lineage from an activated to an unactivated state, thereby suppressing PMN activation by inhibiting IL-8 generation (Figure 7H).

In conclusion, our studies demonstrate that the *ERG*/A20 cascade is an essential safeguard mechanism in ECs, suppressing IL-8 levels to restrict neutrophilic vascular injury. As the loss of *ERG* is observed in chronic lung injury, including ARDS, pulmonary arterial hypertension, and fibrosis (25, 26, 66), our findings will have implications for understanding and therapeutically mitigating PMN function in inflammatory diseases.

### Limitations of the study.

Although our study provides compelling evidence for EC *Erg* as a crucial mechanism in restricting PMN accumulation and activation in the lungs, we acknowledge several caveats. We demonstrated that EC *Erg* deletion also increases the frequency of PMNs in the bone marrow. Whether the reduction in *Erg* in ECs of the bone marrow contributes to regulating PMN mobilization and priming before they reach the lung needs to be determined. Additionally, while we used the lung as a model to assess the impact of *Erg* deletion in ECs on PMN recruitment and activation due to the large capillary surface area, assessing PMN status in other organs may also provide important clues.

We demonstrated that the genetic loss of vascular *ERG* phenocopies PMN infiltration, as observed during pneumonia and in the lungs of patients with ARDS. However, future studies must assess the mechanisms that reduce *ERG* expression in ECs following infections, as well as the signaling cascade that alters PMN activation downstream of CXCR2.

PMNs also undergo reverse transmigration from the tissue to the bone marrow, thereby controlling tissue homeostasis. While we showed that Reparixin and EC-*A20* gene delivery reduced the number of PMNs in the lungs, it remains unclear whether these compounds impact the reverse transmigration and apoptosis of PMNs. Furthermore, the mechanisms by which PMNs upregulate CXCR2 in response to proinflammatory cytokines, such as IL-8, produced by ECs, are not well understood. Reparixin inhibits both CXCR1 and CXCR2 (67, 68). While studies have shown that CXCR2 is the dominant receptor in mice for PMN migration and chemotaxis (21–23, 69), the role of CXCR1 in this mechanism cannot be ruled out. Future studies will be needed to directly assess whether inhibiting IL-8 levels exclusively in ECs using IL-8–blocking antibodies is effective in resolving neutrophilic injury.

PMN transcriptomics varies between homeostasis and inflammation. Understanding the divergent subsets of PMNs that infiltrate upon *Erg* loss in ECs remains to be determined. Therefore, exploring *Erg*-dependent PMN subsets and defining their tissue-driven transcriptional plasticity during *Erg*-mediated pulmonary vascular dysfunction may offer insights into PMN-targeted immunomodulatory strategies to prevent various lung complications.

## Methods

Further information can be found in Supplemental Methods.

### Sex as a biological variable

Our study examined male and female animals, and similar findings are reported for both sexes.

### Knockout and WT mice

*iEC-Erg^–/–^* mice were generated by crossing *Erg^fl/fl^* mice with mice containing the tamoxifen-inducible *Cdh5^cre-ERT2^* promoter driver (70, 71). *Erg^fl/fl^* mice were a gift from Masahiro Iwamoto (University of Maryland School of Medicine, Baltimore, Maryland, USA). *Erg^fl/Cdh5Cre-ERT2^* mice were further crossed with B6.*Cg-Gt(ROSA)26Sor^tm9(CAG-81tdTomato)Hze^*/J to produce R26R*^tdTomato^Erg-cdh5^CreERT2^* mice for converting ECs into tdTomato^+^ ECs (red color) (56, 57). Yoshi Tsukazaki (Louisiana State University Health Science Center at Shreveport) provided Catchup (C57BL/6-*Ly6g^tm2621(Cre-tdTomato)Arte^*) mice. C57BL/6J mouse breeding pairs initially obtained from The Jackson Laboratory were bred and maintained at the University of Illinois Chicago (UIC). Mouse colonies were kept in a pathogen-free housing facility at the UIC. All experiments were performed on C57BL/6J background mice of either sex between 5 and 8 weeks old. All *Erg^fl/Cdh5Cre-ERT2^* mice were generated by backcrossing to the C57BL/6J background for 12 generations (N12).

### Tamoxifen and reparixin treatment

To induce *Erg* deletion, tamoxifen (80 mg/kg, i.p.) was injected into 4- to 5-week-old mice for 5 consecutive days, followed by 1 week of rest to allow for drug washout, as described previously (70–72). A detailed description of this treatment based on the previous work is outlined in the Supplemental Methods.

#### Reparixin treatment.

After 5 days of tamoxifen administration, *Erg^fl/fl^* and *iEC-Erg^–/–^* mice received 30 mg/kg body weight reparixin (i.p.) daily for 5 days. On the 11th day, the lungs were excised and analyzed for mRNA, protein, edema, histology, and flow cytometric analysis.

### PMN RNA-seq and analysis

PMNs were isolated from pooled lung cell suspensions of 3 *Erg^fl/fl^* and *iEC-Erg^–/–^* mice using anti-Ly6G, anti-CD45, and anti-CD31 antibodies. Total RNA was extracted, and RNA integrity was assessed using an Agilent 2100 Bioanalyzer; only samples with a RNA integrity number (RIN) of greater than 8 were used, as described previously (73). Strand-specific libraries were prepared with the TruSeq mRNA library preparation kit (Illumina), and library quality was verified by Bioanalyzer before sequencing on an Illumina NovaSeq 6000 platform (paired-end, 150 bp). Adaptor sequences and low-quality bases were removed using Cutadapt (https://cutadapt.readthedocs.io/en/stable/), and the trimmed reads were aligned to the mouse reference genome (mm10) with HISAT2 (https://github.com/DaehwanKimLab/hisat2). Gene-level read counts were obtained using the featureCounts Subread package (https://subread.sourceforge.net/featureCounts.html). Differential expression analysis was performed using edgeR (https://github.com/OliverVoogd/edgeR) after filtering for low-abundance genes and normalizing counts using the trimmed mean of M values (TMM) method. Differentially expressed genes were identified using a quasi-likelihood *F* test with an FDR-adjusted *P* value of less than 0.05. Pathway enrichment analysis was conducted using Metascape (https://metascape.org/gp/index.html#/main/step1). The top 500 genes with the highest expression in control (*Erg^fl/fl^*) PMNs and their corresponding expression differences in *iEC-Erg^–/–^* PMNs were used as input. Significantly enriched pathways and biological processes were identified based on an FDR-adjusted *P* value of less than 0.05. Data visualization and plots were generated using ggplot2, EnhancedVolcano, and ComplexHeatmap packages in R (https://posit.co/download/rstudio-desktop/).

### PMN adoptive transfer and analysis in the lung and BALF

The lung cell suspension PMNs from control and *iEC-Erg^–/–^* mice were stained with anti-CD45 (PE-cy7), anti-CD31 (APC), and anti-Ly6G (PE) antibodies, after which the PMNs were subsequently sorted by flow cytometry. The sorted PMNs (1 × 10^6^ cells) from donor mice were relabeled with an anti-Ly6G (PE) antibody and adoptively transferred into naive WT mice via i.v. injection. After 4 hours, the recipient animals were euthanized, and their lungs were harvested for flow cytometry, lung injury assessment, and mRNA analysis. For the flow cytometric analysis, the lung cell suspensions from the recipient mice were stained with anti-CD45 (FITC) and anti-Ly6G (BV785) antibodies. Distinct fluorochromes were used for anti-Ly6G (in donor vs. recipient mice) to differentiate the adoptively transferred from the endogenous PMNs clearly. PBS was used as a vehicle control (51).

In a separate experiment, the extravasation of donor PMNs from control and *iEC-Erg^–/–^* mice was analyzed in the BALF of recipient WT mice. For this assay, PMNs from control and *iEC-Erg^–/–^* mice were sorted by flow cytometry, as described in the above methods, using anti-CD45 (FITC) and anti-Ly6G (BV785) antibodies. The sorted PMNs (1 × 10^6^ cells) from donor mice were relabeled with an anti-Ly6G (BV785) antibody and adoptively transferred into WT mice via i.v. injection. After 4 hours, the recipient animals were euthanized, and BALF was obtained and labeled with an anti-Ly6G antibody (PE). FACS analysis was then performed to distinguish between endogenous and transferred PMNs.

### PA-induced neutrophilic lung injury and mouse survival

*PA* was cultured as described previously (74, 75) and described in detail in the Supplemental Methods.

### Human participants

Peripheral blood was collected by venipuncture from (*n* = 3) random healthy volunteers and (*n* = 3) patients with ARDS. Donors or their surrogates provided written informed consent. Blood PMNs were isolated using the MACSxpress Whole Blood Neutrophil Isolation Kit, human (Miltenyi Biotec, 130-104-434). Human lungs from deidentified autopsied patients dying of ARDS or non–lung-related diseases were obtained from the UIC biorepository. The sections were then immunostained with anti-ERG, anti-MPO, and anti-Ly6G antibodies, and DAPI, followed by immunofluorescence imaging using a Zeiss LSM 880 confocal laser scanning microscope, as described previously (49).

### Cell culture

Human pulmonary artery endothelial cells (HPAECs) were cultured in a 0.1% gelatin–coated flask using EBM-2 Endothelial Cell Growth Basal Medium-2 supplemented with growth factors (Lonza, 00190860) and 15% fetal bovine serum (FBS) (Thermo Fisher Scientific, A5256701) (70, 71).

HL-60 cells (human promyelocytic leukemia cells) (ATCC, CCL-240) were grown in suspension with RPMI-1640 medium containing L-glutamine and 25 mM HEPES (Thermo Fisher Scientific, 72400047), supplemented with 15% FBS (Thermo Fisher Scientific, A4766801) and penicillin-streptomycin-amphotericin B (Thermo Fisher Scientific, 15240096), at 37°C and 5% CO_2_. These cells were exposed to 1.3% DMSO in RPMI-1640 complete media for differentiation and cultured for 6 days. Differentiated cells were then used for experiments.

### Bacteria

GFP-tagged *PA* (GFP-PA01 strain) was used to induce lung injury in mice, as previously described (51, 74).

### Quantitative real-time PCR analysis

Total RNA was isolated from whole lungs or sorted PMNs of indicated mice using TRIzol reagent (Invitrogen) or RNeasy Mini Kit (Qiagen, 74104) according to the manufacturers’ instructions. RNA was quantified using a Biodrop, and reverse transcription reaction was carried out as per published protocols (70). The primers used are listed in Supplemental Table 1.

### Western blotting

Tissues or cells lysed in RIPA buffer (Sigma-Aldrich, R0278) were immunoblotted as described previously (70).

### FACS analysis

FACS analysis was performed in the indicated lungs and spleen as described previously (70). A detailed method is given in Supplemental Methods.

### Immunofluorescence and image analysis

Lungs harvested from the indicated mice were fixed in 4% paraformaldehyde (PFA) for 4 hours, after which they were transferred into a 30% sucrose solution for 24 hours and then embedded in OCT. FITC-IB4 (50 mg/100 μL PBS) was injected into each mouse through the tail vein 2 hours before harvesting the lungs. The lungs were cryosectioned (4 μm thickness) and immunostained using anti-ERG or anti–VE-cadherin primary antibodies, followed by appropriate secondary antibodies and DAPI. For immunostaining of ECs, cells were fixed in 2% PFA for 10 minutes, followed by permeabilization with 0.1% Triton X-100 for 2 minutes. Cells were stained with the indicated antibodies and appropriately labeled secondary antibodies, followed by DAPI. The cells images were acquired with an inverted laser-scanning confocal microscope (LSM 880, Carl Zeiss Microscopy) using the Zeiss LSM software. All image analyses were quantified by measuring fluorescence intensity using ImageJ software (70, 76).

### Lung intravital imaging and PMN assessment

Lung intravital imaging was performed as described previously (77, 78). The detailed description is provided in Supplemental Methods.

### BALF histology and ROS imaging

BALF was obtained as described previously (51). The detailed description is provided in Supplemental Methods.

### A20 gene delivery to the mouse lungs

Vector and A20 cDNA were delivered in control and *iEC-Erg^–/–^* mice using cationic liposomes prepared by dissolving dimethyl dioctadecyl ammonium bromide (MP Biomedicals, 150939) and cholesterol (Calbiochem, 228111) in chloroform, as described previously (73, 79). The detailed description is provided in Supplemental Methods.

### Transfections

HPAECs were transfected with control (siCont) or *ERG* siRNA (siERG) targeting Erg-1/2/3 (Santa Cruz Biotechnology, sc-35334) to deplete ERG. Briefly, ECs at 60%–80% confluence were transfected with the indicated siRNA using siRNA Transfection Reagent (Santa Cruz Biotechnology, sc-29528) (70). Control siRNA (ON-TARGETplus Non-targeting Pool; D-001810-10, Dharmacon) was used in all experiments. The cells were assessed 72 hours after transfection. To rescue A20 expression, ECs transfected with siERG for 48 hours were retransfected with A20 WT or A20-DUB mutant cDNA or control vector using Fugene HD Transfection Reagent (Promega, E2311). These cells were then used 24 hours after cDNA transfection (76). Immunoblotting and qPCR were performed to confirm *ERG* depletion or cDNA expression.

### EC–HL-60 coculture

Differentiated HL-60 cells were cocultured with ECs transfected with control or *ERG* siRNA. After 24 hours, the HL-60 cell suspension was aspirated and collected in a separate tube. The remaining EC-adherent PMNs were isolated after trypsinization using a MACSxpress Whole Blood Neutrophil Isolation Kit (Miltenyi Biotec, 130-104-434) according to the manufacturer’s protocols. The HL-60 cells were then subjected to RNA and protein studies as indicated. HL-60 cells adhered to control or siERG ECs were imaged using ECHO Brightfield Imaging at ×40 optical magnification.

### ELISA

MIP2α from the blood of *Erg^fl/fl^* and *iEC-Erg^–/–^* mice (Mouse CXCL2 ELISA Kit, Proteintech, KE10022), and in vitro IL-8 release from cell supernatants were assessed using commercially available assay kits (Recombinant Human IL-8/CXCL8 Protein, R&D Systems, 208-IL-010/CF), according to the manufacturers’ instructions.

### MPO assay and immunostaining

MPO activity in *Erg^fl/fl^* and *iEC-Erg^–/–^* mouse lung lysates was assayed colorimetrically, as described previously (80). The data are expressed as units of MPO activity per gram of lung tissue, where 1 unit was defined as the change in OD at 460 nm.

For MPO immunostaining, *Erg^fl/fl^* and *iEC-Erg^–/–^* mice were euthanized and after perfusing them with PBS, lungs were harvested, fixed, embedded in paraffin, and sectioned. The paraffin-embedded lung sections were then deparaffinized, and antigen retrieval was performed, following which sections were permeabilized with 0.2% Tween 20 in PBS. After 20 minutes, sections were washed 3 times with wash buffer (PBS containing 0.05% Tween 20), and immunostained with an anti-MPO antibody (Abcam, ab208670; 1:1000 dilution) and DAPI (1:1000 dilution), followed by staining with an appropriate secondary antibody. Images were acquired using a 63× objective and a Zeiss LSM 880 confocal microscope. For quantitative analysis, the images were converted into an 8-bit format, color channels were split, and multiple areas in a given lung’s sections were observed and MPO^+^ cells were counted in ImageJ. The plot shows MPO^+^ cells, which represent mean MPO fluorescence intensity/region of interest, pooled together from 3 lungs/group performed independently.

In Figure 4D, lungs from the indicated group of mice were fixed, sectioned, and permeabilized as above. Permeabilized lung sections were immunostained with anti-MPO antibody (Abcam, ab208670; 1:1000 dilution), anti–VE-cadherin (Santa Cruz Biotechnology, sc-9989; 1:50 dilution), and DAPI (1:1000), followed by staining with the appropriate secondary antibodies. MPO^+^ cells, quantified as above, were pooled from 6 lungs/group.

### PA culture

*PA* was cultured as described previously (74, 75). The detailed description is provided in Supplemental Methods.

### Lung histology

ALI severity was calculated using the method previously described (74, 75). Briefly, histology of the lung was used to score for (i) PMNs in the alveolar space, (ii) PMNs in the interstitial space, (iii) hyaline membranes, (iv) proteinaceous debris filling the airspace, and (v) alveolar septal thickening. Each item (i–v) was given a score between 0 and 4. A minimum score of zero would represent a healthy lung, and a maximum score of 4 would represent a lung with severe ALI.

### Assessment of lung vascular injury

Lung wet-dry weight ratios were determined to quantify lung vascular injury, as described previously (70, 75).

### SeqChIP assay

SeqChIP was employed to assess the binding of *ERG* and NF-κB to the *A20* promoter sequentially, as described previously (70, 79, 81, 82). The detailed description is provided in Supplemental Methods.

### Analysis of publicly available neutrophil single-cell RNA-seq data

To compare neutrophil transcriptional profiles with those identified in this study, we analyzed a publicly available single-cell RNA-seq dataset from BALF of healthy participants and patients with COVID-19 (NCBI GEO accession number GSE145926) (83). Raw count matrices were processed using Seurat (v5.1). Data were normalized using the LogNormalize method, and the top 2,000 variable genes were identified with the “vst” selection method. Mitochondrial gene content (percent.mt) was regressed out during scaling. Principal component analysis (PCA) was performed using the top 50 principal components. Cell clustering was performed with 50 dimensions and a resolution of 1.2. Neutrophil clusters were identified based on canonical marker expression, including *FCGR3B*, *CXCR2*, *CD33*, *CXCR4*, and *IL1B*. These clusters were isolated using the Subset function to generate datasets specific to neutrophils. Each dataset was further log-normalized and reclustered at a resolution of 0.05 to obtain one representative cluster per patient. Datasets from individual patients were then merged using the merge function to allow group-wise comparison between healthy and COVID-19 samples. Data visualization was performed using ggplot2 in R.

### Analysis of publicly available EC database

To analyze *ERG* expression in different cell types, Tabula muris (public database) was used (http://tabula-muris.ds.czbiohub.org/ Accessed July, 2024.).

### Software and algorithms

The GEO database accession number was GSE120000. FlowJo 10.5.3 was used to analyze FACS plots. Zen Lite was used in confocal imaging and processing (Zeiss Inc.). Biorender was used to make schematics and illustrations.

### Statistics

Statistical analysis was performed using Prism version 8.0 (Graph Pad Software). Comparisons between 2 groups were analyzed using an unpaired, 2-tailed Student’s *t* test. Comparisons among 3 or more groups were performed using ANOVA, followed by Tukey’s multiple-comparison post hoc test. Data are expressed as mean ± standard deviation (SD) from at least 3 independent experiments. Wilcoxon’s signed-rank test was used for analysis of the publicly available dataset (healthy and COVID-19 patients). All the statistical significance details are indicated in the respective figure legends. A *P* value of less than 0.05 was considered significant.

### Study approval

All procedures were performed in accordance with the NIH *Guide for the Care and Use of Laboratory Animals* (National Academies Press, 2011). The Institutional Animal Care and Use Committee of the UIC approved all animal models and experiments used in this study, approval no. IACUC-21-200. The UIC’s Institutional Review Board approved human blood sample collection and experiments.

### Data availability

Data and relevant information will be available directly from the corresponding author. The raw RNA-seq data can be found in the NCBI GEO dataset GSE310680.

### Inclusion and diversity

We support inclusive, diverse, and equitable research conduct.

## Author contributions

VV and DM designed the study, analyzed the data, prepared figures, and wrote the manuscript. VV, VABR, JCJ, and MA performed mouse experiments. VV, VABR, SB, and MA performed immunohistochemistry of lung tissues and FACS analysis of PMNs. MZA and MA performed an in silico analysis of transcription factor binding motifs and a ChIP assay. SD obtained human samples and edited the manuscript. VABR and VP performed RNA-seq data analysis. YT performed intravital lung imaging. SP provided key scientific inputs and edited the manuscript. DM acquired funding. All authors edited and approved the manuscript.

## Funding support

This work is the result of NIH funding, in whole or in part, and is subject to the NIH Public Access Policy. Through acceptance of this federal funding, the NIH has been given a right to make the work publicly available in PubMed Central.

- NIH grants P01-HL151327, P01-HL160469, R01-HL137169, R01-155941, R01-HL084153, and R01-HL165263.

## Supplementary Material

Supplemental data

Unedited blot and gel images

Supporting data values

## Version 1 03/05/2026

In-Press Preview

## Version 2 04/08/2026

Electronic publication
