# Supplementary material for: ERG-deficient endothelium identifies IL-8/CXCR2 axis as a therapeutic target for resolving neutrophilic lung vascular injury
Source: JCI Insight. 2026 Mar 5;11(7):e195989. doi: 10.1172/jci.insight.195989 (PMC13134714; doi:10.1172/jci.insight.195989)
Supplement: Supplemental data [file jciinsight-11-195989-s279.pdf]

Supplementary Figure 1

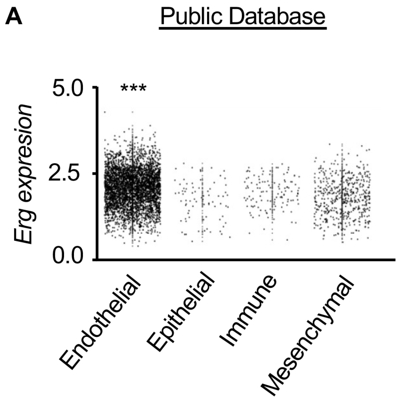

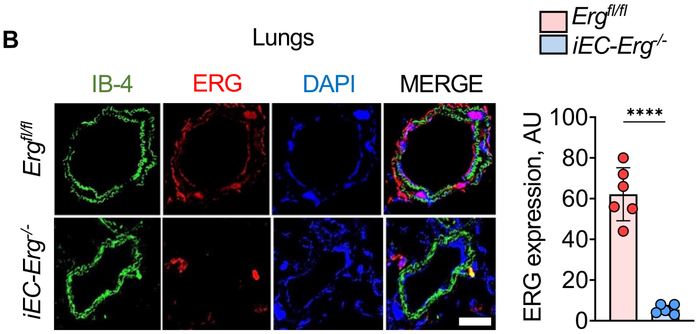

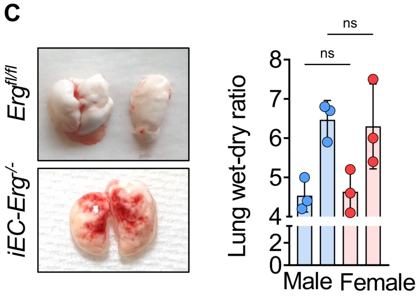

Supplementary Figure 2

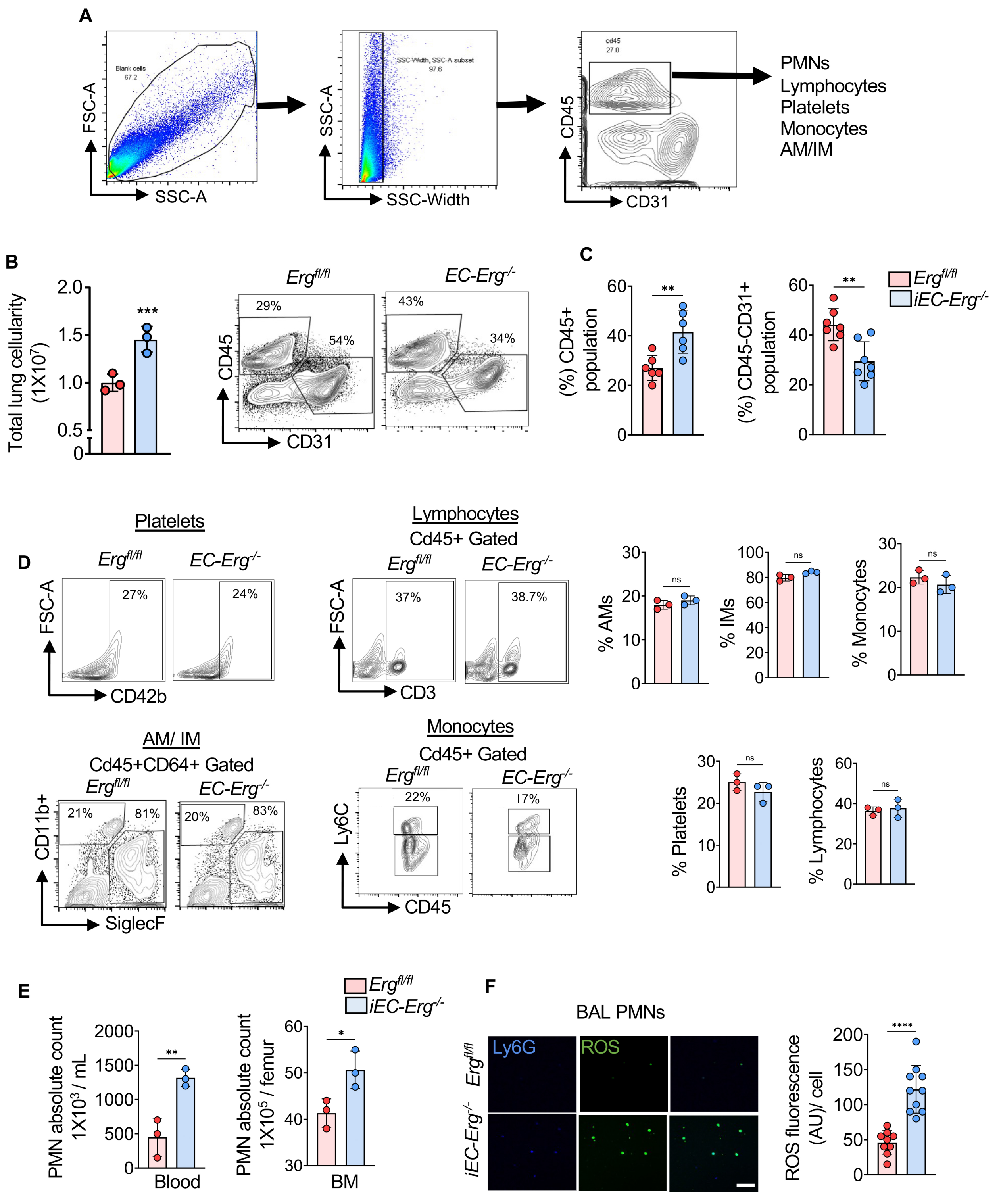

Supplementary Figure 3

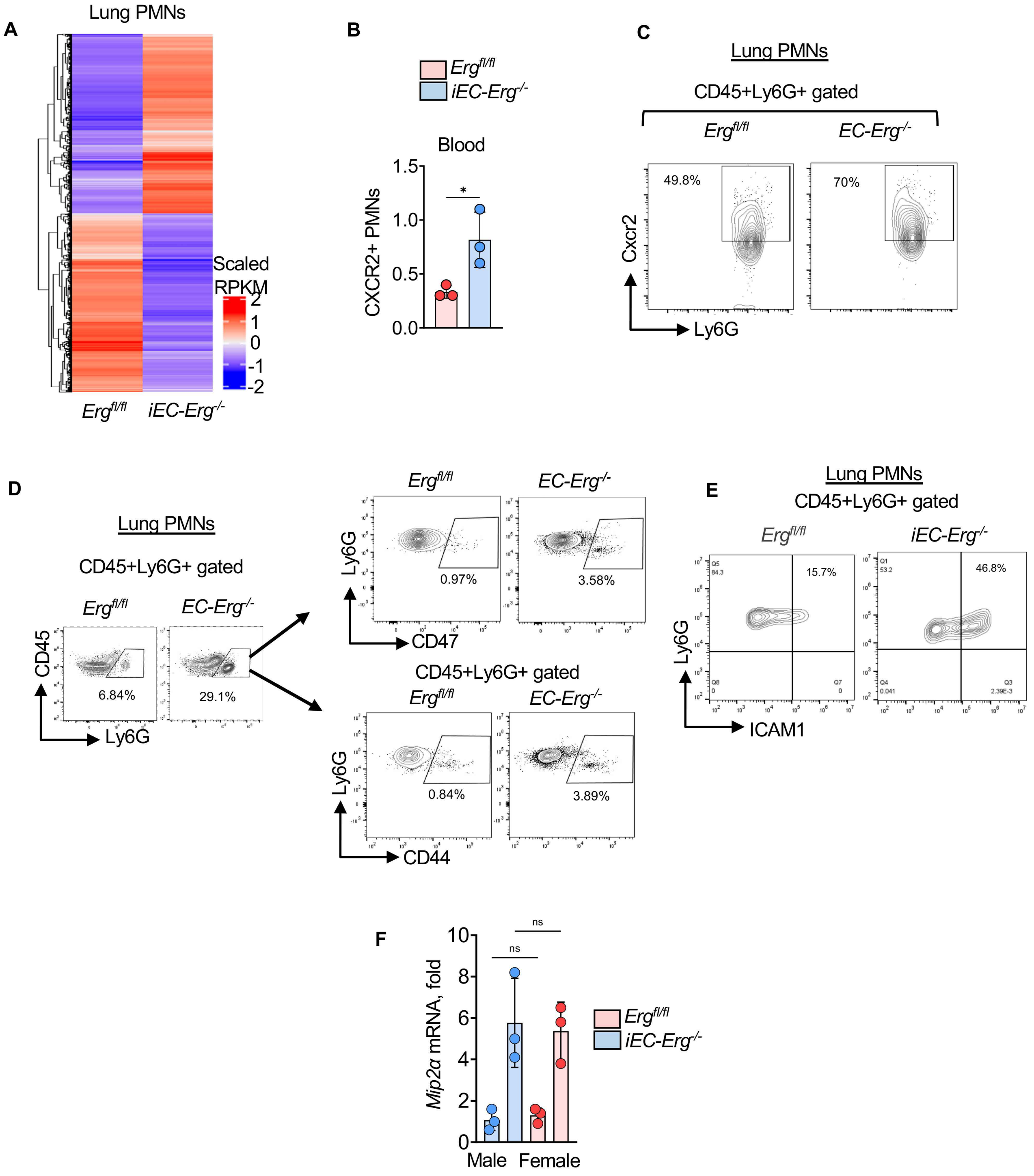

Supplementary Figure 4

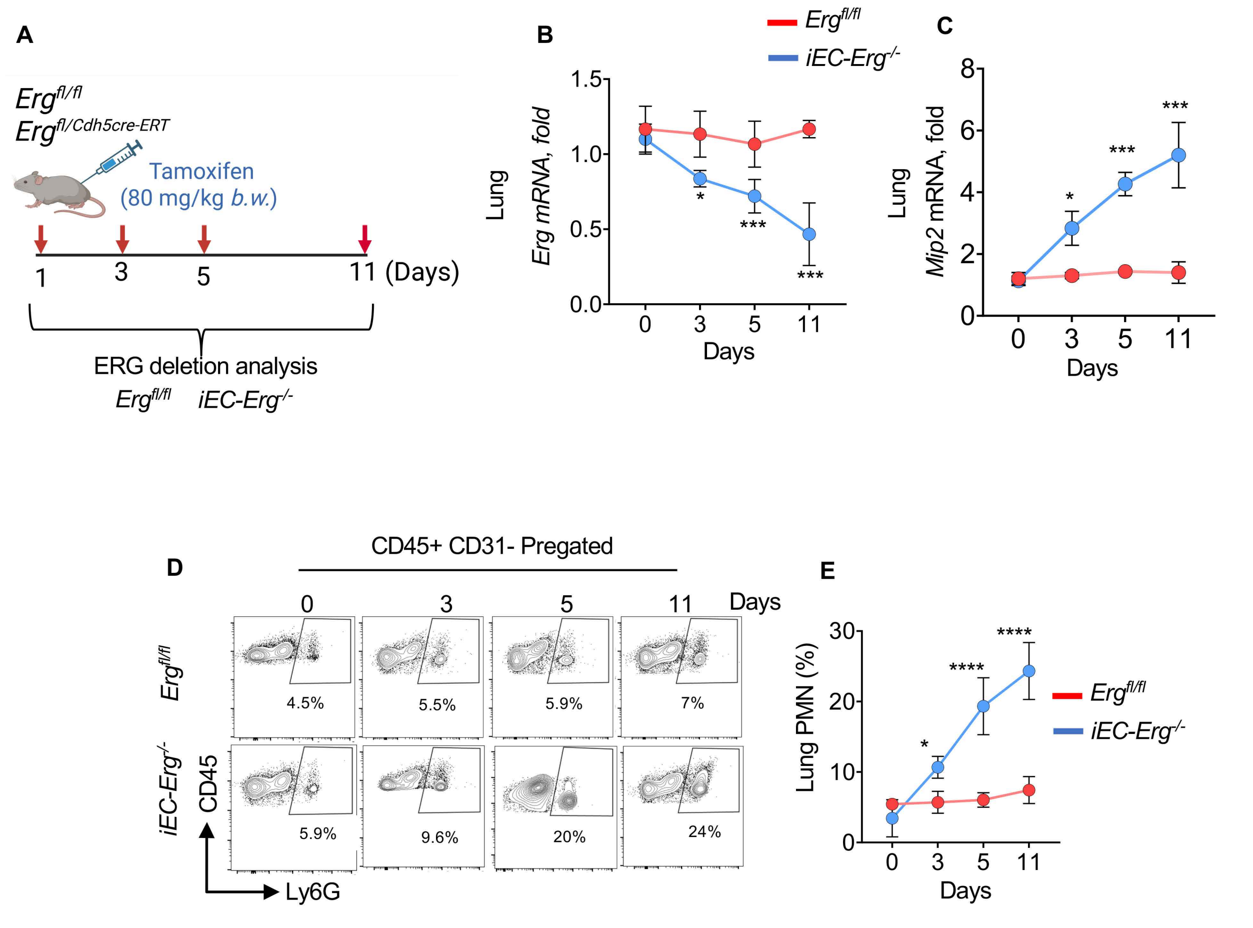

Supplementary Figure 5

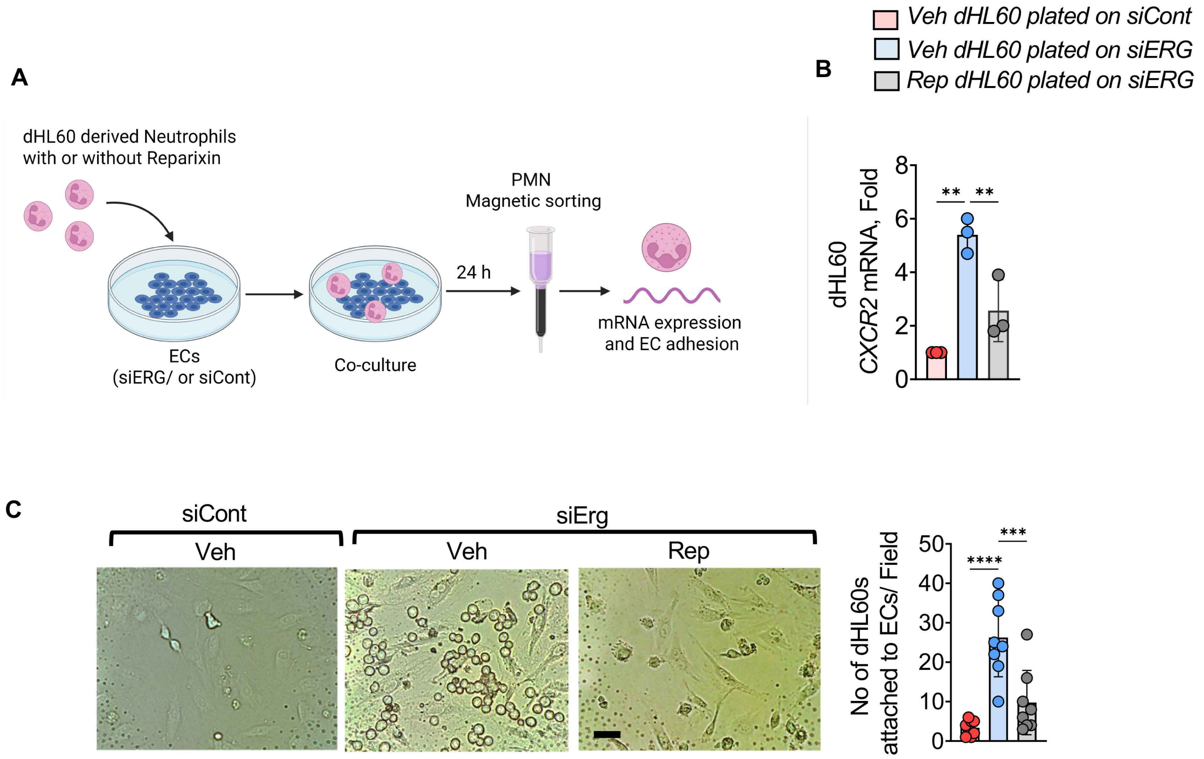

Supplementary Figure 6

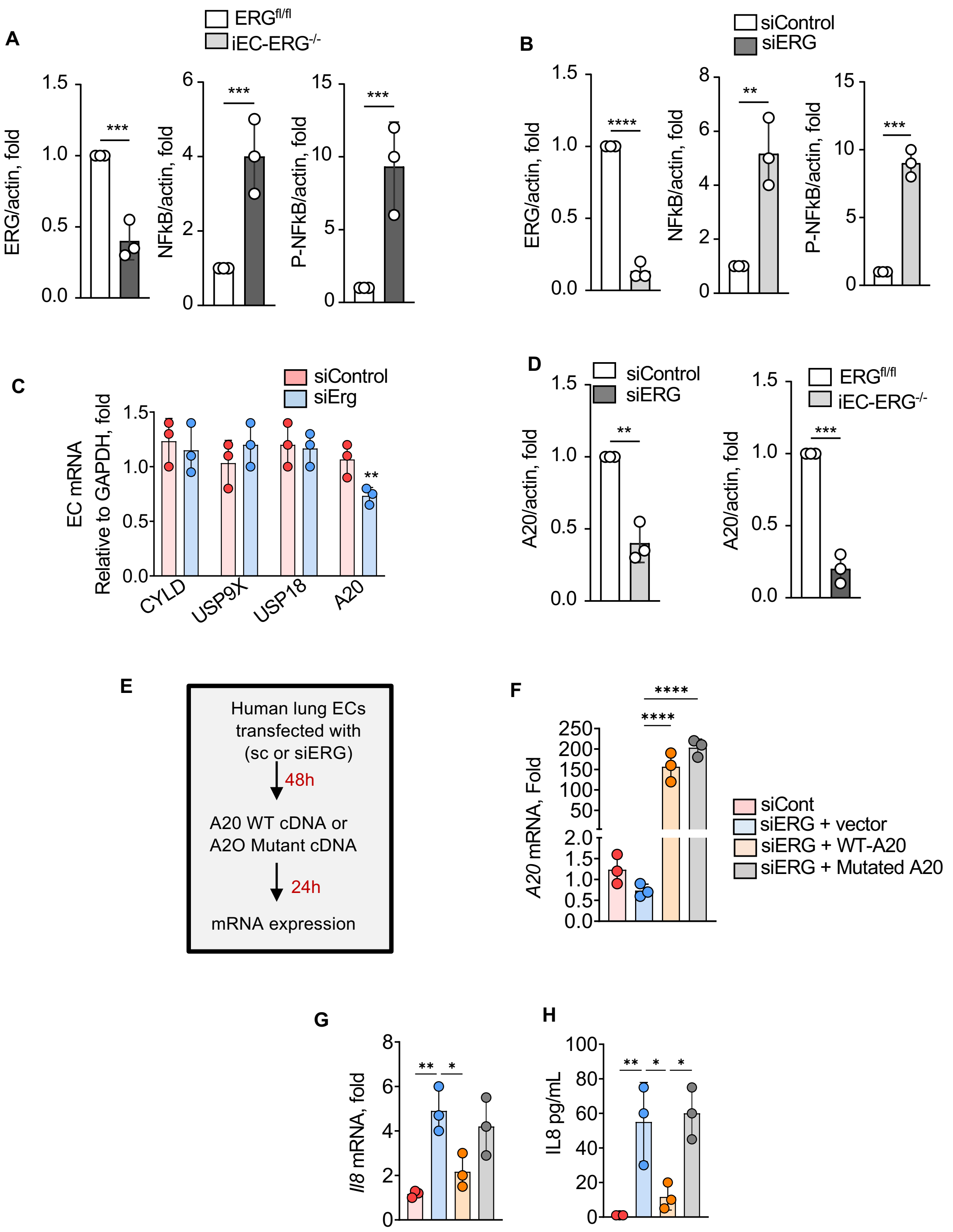

Supplementary Figure 7

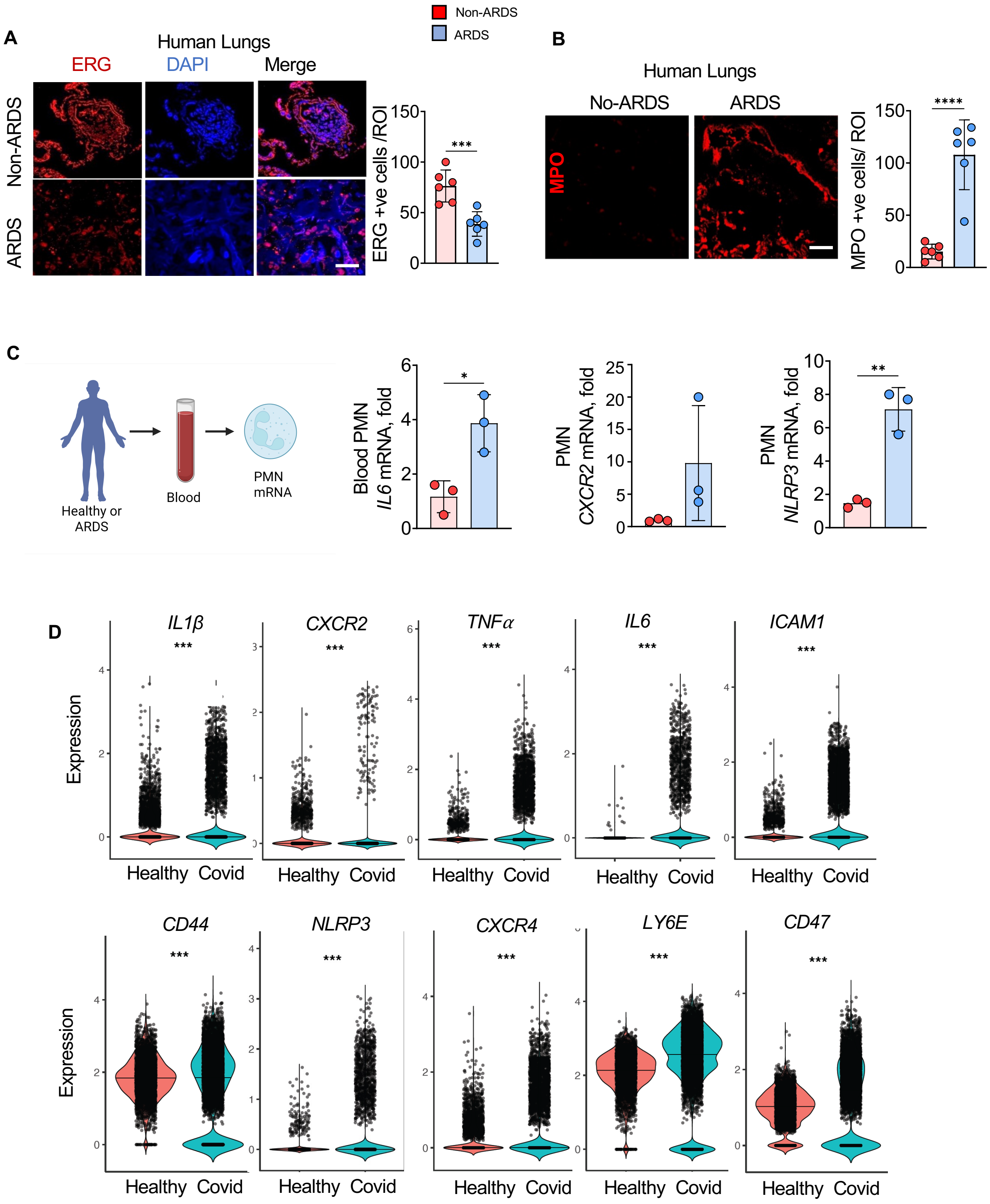

Supplementary Figure Legends:

Supplementary Figure 1. Role of endothelial *ERG* expression in regulating the vascular niche. (A) *ERG* levels in lung cells, as determined using publicly available data (Tabula Muris). (**B**) Isolectin-IB4 and *Erg* staining in *Erg*$^{fl/fl}$ and *iEC-Erg*$^{-/-}$ lungs. The left shows a representative image, while the right shows the quantitation of *Erg* in IB4+ vessels. Scale bar = 20 µm. GAPDH was used as an internal control. **(C)** Gross morphology of *Erg*$^{fl/fl}$ and *iEC-Erg*$^{-/-}$ lungs (left) and lung edema (right). Data represented as ±SD. Wilcoxon test was used for Fig. A. Unpaired Student's T-test (for Fig. B, C, and D). P***<0.001, ****<0.0001, ns – non significant.

Supplementary Figure 2. Validation of *iEC-Erg* role in regulating neutrophil functions. (A) Gating strategy after labeling lung cells with CD31, CD45, SiglecF, CD11b, CD3, and CD42b antibodies. (**B-C**) A representative FACS plot of total lung cellularity, lung CD45+ and CD45-CD31+ populations in indicated mice (B) while C shows the quantification. (**D**) FACS plot and quantitation of alveolar macrophages, monocytes, lymphocytes, and platelets in the indicated mice. (**E**) PMNs absolute number in the blood and bone marrow of *Erg*$^{fl/fl}$ and *iEC-Erg*$^{-/-}$ mice. (**F**)) ROS levels in BAL-PMNs from indicated mice after staining with Ly6G antibody and H2-DCFDA. A representative image is shown on the right, while the left shows quantification. Data represented as ±SD. Unpaired Student's t-tests (all plots). P*<0.05, **<0.01, ****<0.0001, ns – non significant.

Supplementary Figure 3. *iEC-Erg* loss activates neutrophil CXCR2. (A) Heatmap of lung neutrophils isolated from the indicated lungs. **(B-C)** CXCR2 cell-surface expression in blood **(B)** blood **(C)** and lung PMNs. **(D)** CD44 and CD47 expression on the surface of PMNs. **(E)** lung PMN ICAM1 expression. **(F)** Lung *Mip2a* mRNA levels in the indicated group

were normalized using GAPDH. Data represented as ±SD. Unpaired Students' t-tests. P *<0.05, ns – non significant.

Supplementary Figure 4. *iEC-Erg* deletion time course and its influence on PMN accumulation in lungs. **(A)** Schematics of *iEC-Erg* deletion time course. **(B)** Lung *Erg* mRNA in indicated mice taking GAPDH as control (n=6). **(C)** Lung *Mip2α* mRNA taking GAPDH as controls (n=3). **(D)** A representative FACS plot **(E)** and PMNs analysis (n=3). Unpaired Student's t-tests (all plots). P*<0.05, ***<0.001****<0.0001.

Supplementary Figure 5. Reparixin blocks IL8-induced PMN adhesion (A) Schematics showing EC and dHL60 co-culture experiment. (**B**) CXCR2 mRNA expression normalized against GAPDH, n=3. (**C**) Quantitation of HL60 cell adhesion on ECs. The left shows the representative image, while the right shows the quantification of the adhered dHL60 number. Data represented as ±SD. One-way ANOVA followed by Tukey's multiple comparison tests (all plots). P**<0.01, ***<0.001****<0.0001.

Supplementary Figure 6. *ERG* deletion induces IL8 secretion in human endothelial cells. (A-B) Densitometric quantification of immunoblots from Figure 5B. **(C)** mRNA expression of indicated genes from control and *ERG*-depleted ECs normalized against GAPDH. **(D)** Densitometric quantification of immunoblots for Fig. 5D-E. **(E)** Schematics of A20 cDNA overexpression in *ERG-depleted* ECs. **(F-G)** mRNA expression of A20 and IL8. **(H)** ELISA quantification of IL8 in conditioned media of indicated ECs. Data represented as ±SD. Unpaired Students' t-tests (for Figure A-D). One-way ANOVA followed by Tukey's multiple comparison tests (for Figure F-H). P*<0.05, **<0.01, ***<0.001, ****<0.0001.

Supplementary Figure 7: *ERG* expression and neutrophilic injury in ARDS and COVID patients. (A) Representative image of *ERG* expression (left) and quantitation (right) in ARDS and

Non-ARDS human lungs. **(B)** MPO activity in normal and ARDS human lungs. The left shows a representative image, while the right shows the quantification. Scale bar = 40 μm. **(C)** mRNA expression of indicated genes in human peripheral blood neutrophils from normal subjects and ARDS patients (n=3). **(D)** PMN gene expression analysis from publicly available data of bronchoalveolar lavage fluid from healthy and COVID-19 patients. Data represented as ±SD. Unpaired Student's t-tests (all plots) along with Welch's Correction were used. Wilcoxon test was used for Fig D. P*<0.05, **<0.01, ***<0.001, ****<0.0001.

Supplemental Methods:

Antibodies and Reagents

Erg-1/2/3 siRNA (h) (Santa Cruz Cat # sc-35333), SiRNA Transfection Medium (Santacruz Cat # sc-36868), Erg-1/2/3 Antibody (D-3) (Santa Cruz Cat # sc-271048), A20/TNFAIP3 Antibody (59A426) (Novus biologicals, Cat # NBP1-77533SS), SiRNA Transfection Reagent (Santa Cruz Cat # sc-29528), Reparixin MCE MedChem Express (Cat # HY-15251), Ly6G - BV785 flow antibody (Biolegend Cat #127645), Ly6G-PE, Thermofisher, Lipopolysaccharide (Sigma Aldrich Cat #2630), CD11c- PE/APC/FITC, Biolegend (Cat # 117307, 117310, 117305), CD11b APC/APC CY7, Biolegend (Cat # 101212, 101226), CD64- PECY7/APC, Biolegend (Cat # 139306, 139306), CD45- APC FIRE 750 / FITC, Biolegend, (Cat # 103154, 103108), Cholesterol (Calbiochem Cat # 228111), Trizol Life Technologies (Cat # 15596-026), Rabbit monoclonal anti-phospho- NFκB p65(S536, 93H1) (Cell Signaling Cat # 3033S). Protein A Agarose/Salmon Sperm DNA, Millipore Sigma (Cat # 16-157), Phospho-NF-κB p65 (Ser536) (93H1) Rabbit mAb (Cell signalling Cat #3033), PROMEGA EXPRESS (E2311), FuGENE® HD Transfection Reagent (Cat # E2311), VE-cadherin (F-8) antibody (SantaCruz, Cat # sc-9989), Mouse CXCL2 ELISA Kit (Proteintech Cat # KE10022), Recombinant Human IL-8/CXCL8 Protein (R&D Cat #208-IL-010/CF), CD184 (CXCR4) Monoclonal Antibody (2B11), PE-Cyanine7, eBioscience™ (Thermofisher Cat# # 25-9991-80), LONZA-EBMTM-2 Endothelial Cell Growth Basal Medium-2 (CC-3156), Alexa Fluor® 488 anti-mouse CD31 (PECAM-1) Antibody, Human IL8 ELISA strip (Signosis Cat# CusEA1001), MACSxpress® Whole Blood Neutrophil Isolation Kit (Abcam Cat# 130-104-434).

Tamoxifen and Reparixin treatment

To induce *Erg* deletion, tamoxifen (80 mg/kg, *i.p.*) was injected into 4-5 week old mice for five consecutive days, followed by a week of rest to allow for drug washout, as described previously (1-3). Briefly, 20 mg tamoxifen (Sigma, Cat No. T5648) was suspended in 1 mL of corn oil (Sigma, Cat No. C8267). The mixture was vortexed at full speed for 30 minutes, after which it was wrapped in aluminum foil and placed in an orbital shaker overnight. The drug was filtered using a 0.2 µm filter before being injected into the animal. All experiments were performed on 6-8 week-old mice weighing 20-25 g. On the 11th day, the lungs were excised and analyzed. To determine the time course of *Erg* deletion, lungs were isolated after 1, 3, and 5 doses of Tamoxifen administration. After each indicated dose, the lungs from the specific groups were excised and analyzed to check *Erg* deletion and FACS.

***Pseudomonas aeruginosa*-induced neutrophilic lung injury and mouse survival.**

PA was cultured as described previously (4, 5). Briefly, the anesthetized mice receive 1×10^5 CFU PA dissolved in 50 µL PBS/mouse through the endotracheal route. Mice were sacrificed, and the lungs were excised for protein, RNA, and FACS analysis at the indicated times.

For survival studies, four groups were made after Tamoxifen administration and washout. *Erg* flox, sham control, *iEC-Erg* vector, *iEC-Erg* -A20, and *iEC-Erg* -Reparixin. PA (1×10^5 CFU *i.t.*) dissolved in 50 µL PBS/mouse was instilled into mice, and mouse survival was assessed every 6-12 hours. Reparixin (30 mg/kg body weight) was administered daily for 5 days or until the animal's survival was achieved. Similarly, A20 cDNA liposomes were administered *i.v.* until the animal survived. The animals' weight and death were evaluated to determine the survival curve using the Kaplan-Meier graph.

FACS analysis

FACS analysis was performed in the indicated lungs as described (2). Briefly, after mincing, tissues were enzymatically digested with 1 mg/mL collagenase A (Roche, New York, NY) for 50 min at 37°C, forced through a metal cannula, and passed through a 75-mm nylon filter to obtain single-cell suspensions. The red blood cells were lysed using lysis buffer, and the cell suspensions were washed with FACS buffer. The bone marrow was flushed and then passed through a 0.45 μm filter to obtain a cell suspension. Cells were re-suspended in FACS buffer, and after Fc block with FcgRIII/ II antibody for 30 minutes, cells were labeled with indicated antibodies as a cocktail (anti-CD31, anti-CD45, anti-CD64, anti-CD11c, anti-CD11b, anti-Gr1 (anti-Ly6G), anti-SiglecF, anti-CD62P, and anti-CD3), for 30 minutes on ice. Samples were washed and analyzed using a CytoFLEX LX Flow Cytometer (Beckman Coulter), and data were processed using FlowJo™ Software v10.10. All antibodies used for flow cytometry were specific to mouse antigens.

Lung intravital imaging and neutrophil assessment

Lung intravital imaging was performed as described (6, 7). Briefly, *td-Tomato-Erg*$^{fl/fl}$ and *td-Tomato-iEC-Erg*$^{-/-}$ mice were injected with ketamine (10 mg/ml) and xylazine (2.5 mg/ml) at 40-80 mg/kg body weight (for ketamine) and 10-20 mg/kg body weight (for xylazine). SeTau647(SETA BioMedicals)-labeled CD31 antibody (25 μg/mice) (clone 390; Biolegend) and BV421-labeled Ly6G (Clone 1A8; Biolegend) antibody (10 μg/mice) were retro-orbitally injected right before the surgery and lung intravital imaging to stain intravascular PMNs and lung microvascular structures. A resonance-scanning two-photon microscope (Ultima Multiphoton Microscopes, Bruker) with an Olympus XLUMPlanFL N 20x (NA 1.00) and Immersion oil (Immersol W (2010); Carl Zeiss) were employed to collect multi-color images (Dichroic mirror;

775 extended pass filter (775 LP; Bruker), IR blocking filter; 770 short pass filter (770 SP; Bruker), Emission filter; 460/50 nm for BV421, 595/60 nm for tomato (Bruker) and 708/75 nm for SeTau647 (FF01-708/75-25; Semrock) with 960 nm excitation at video rate. Motion artifacts were stabilized using computer vision algorithms based on image processing of lung microscopic images. ImageJ, Origin (OriginLab), and customized LabVIEW programs (National Instruments) were used to quantify PMN dynamics.

BALF histology and ROS imaging

Bronchoalveolar lavage fluid (BALF) was performed as described previously (8). Briefly, after sacrificing the mice, a tracheotomy was performed, followed by the collection of BAL using an 18-gauge blunt needle. The BAL fluid was cytospun by centrifuging at 2000 rpm for 10 min, followed by Hematoxylin and Eosin (H&E) staining to quantify neutrophils. The sections were imaged at 20X magnification using an ECHO Brightfield Imaging at 40X optical magnification.

The BAL cells plated on a 30 mm cover dish for live ROS assessment were treated with the cell-permeant 2',7'-dichlorodihydrofluorescein diacetate (H2DCFDA) (Thermofisher #D399). After 30 minutes, the cells were washed and immunostained with anti-Ly6G antibody. After 30 minutes, immunofluorescence was determined by confocal imaging. ROS fluorescence intensity was measured using ImageJ software.

A20 gene delivery to the mouse lungs

Vector and A20 cDNA were delivered in control and *Erg* -deleted mice using cationic liposomes as described previously (9). Briefly, A lipid layer was formed by evaporating chloroform using a rotavapor system (105 rpm for 15–20 min at 37°C), dissolved in 5% glucose, and extracted by sonicating the solution for 1 h at 37°C. Liposomes were filtered using a 0.45-micron filter, after which cDNA (vector or A20) was added slowly with vortexing to avoid the precipitation of

cDNA. The control or A20 cDNA-loaded liposomes (50 mg in 100 μL/mouse) were administered retro-orbitally into the mouse. After 48 h, the lungs were excised and analyzed for edema, protein, gene expression, and FACS analysis.

***Pseudomonas aeruginosa* culture**

PA was cultured as described previously (4, 5). Briefly, PA from glycerol-preserved stock was streaked on ampicillin-selective (HiMedia Laboratories LLC, United States) Luria–Bertani (LB) agar plates and incubated overnight at 37°C. Single colonies were picked and inoculated overnight (~17 h) in 250 ml LB broth containing ampicillin (100 μg/ml) at 37°C. For the standard plate count method, 1 ml of the bacterial culture from the broth was serially diluted with sterile phosphate buffer up to 1×10^{10} dilutions. Each dilution plate was spread-plated onto ampicillin-selective (100 μg/ml) agar plates to obtain countable bacterial colonies. The 1×10^{5} CFU/25mL was calculated using the following formula: CFU/ml = (no. of colonies x dilution factor)/volume of the culture plate.

Sequential Chromatin Immunoprecipitation Assay:

Sequential chromatin immunoprecipitation (SeqChIP) was employed to assess the binding of *ERG* and NFκB to the A20 promoter sequentially, as described earlier (2, 10-12). Briefly, ECs transfected with scrambled or *ERG* siRNA were stimulated with LPS (1 mg/ml). Formaldehyde cross-linked protein-DNA complexes (100–125 mg) were immunoprecipitated with the anti-ERG antibody, while normal rabbit IgG was used as an antibody control. Protein A/G agarose beads were used to pull down the antibody-chromatin complex. Subsequently, agarose beads were resuspended in the dilution buffer, and the second anti-NFκB antibody was added. Protein-associated DNA was recovered by incubating at 65 °C, purified by phenol extraction, and precipitated with ethanol as described. The promoter region of A20, which corresponds to a 140bp

fragment containing *ERG* and NFκB binding sites, was quantified by real-time q-PCR. The DNA from the input (20-40 mg protein-DNA complexes) was used as an internal control. The experiments were performed three times with independent samples. The following primers were used for *ERG,* which spans -300 kb and *NFκB* which spans -270 kb **(Supplementary Table 2)**.

References:

1. Balaji Ragunathrao VA, Anwar M, Akhter MZ, Chavez A, Mao Y, Natarajan V, Lakshmikanthan S, Chrzanowska-Wodnicka M, Dudek AZ, Claesson-Welsh L, et al. Sphingosine-1-Phosphate Receptor 1 Activity Promotes Tumor Growth by Amplifying VEGF-VEGFR2 Angiogenic Signaling. *Cell Rep.* 2019;29(11):3472-87 e4.
2. Akhter MZ, Chandra Joshi J, Balaji Ragunathrao VA, Maienschein-Cline M, Proia RL, Malik AB, and Mehta D. Programming to S1PR1(+) Endothelial Cells Promotes Restoration of Vascular Integrity. *Circ Res.* 2021;129(2):221-36.
3. Akhter MZ, Yazbeck P, Tauseef M, Anwar M, Hossen F, Datta S, Vellingiri V, Chandra Joshi J, Toth PT, Srivastava N, et al. FAK regulates tension transmission to the nucleus and endothelial transcriptome independent of kinase activity. *Cell Rep.* 2024;43(6):114297.
4. Rayees S, Joshi JC, Joshi B, Vellingiri V, Banerjee S, and Mehta D. Protease-activated receptor 2 promotes clearance of Pseudomonas aeruginosa infection by inducing cAMP-Rac1 signaling in alveolar macrophages. *Front Pharmacol.* 2022;13(874197.
5. Joshi JC, Joshi B, Zhang C, Banerjee S, Vellingiri V, Raghunathrao VAB, Zhang L, Amin R, Song Y, and Mehta D. RGS2 is an innate immune checkpoint for TLR4 and Galphaq-mediated IFNgamma generation and lung injury. *bioRxiv.* 2023.
6. Tsukasaki Y, Toth PT, Davoodi-Bojd E, Rehman J, and Malik AB. Quantitative Pulmonary Neutrophil Dynamics Using Computer-Vision Stabilized Intravital Imaging. *Am J Respir Cell Mol Biol.* 2022;66(1):12-22.
7. Looney MR, and Headley MB. Live imaging of the pulmonary immune environment. *Cell Immunol.* 2020;350(103862.
8. Joshi JC, Joshi B, Rochford I, Rayees S, Akhter MZ, Baweja S, Chava KR, Tauseef M, Abdelkarim H, Natarajan V, et al. SPHK2-Generated S1P in CD11b(+) Macrophages Blocks STING to Suppress the Inflammatory Function of Alveolar Macrophages. *Cell Rep.* 2020;30(12):4096-109 e5.
9. Joshi JC, Joshi B, Zhang C, Banerjee S, Vellingiri V, Raghunathrao VAB, Anwar M, Rokade TP, Zhang L, Amin R, et al. RGS2 is an innate immune checkpoint for suppressing Galphaq-mediated IFNgamma generation and lung injury. *iScience.* 2025;28(2):111878.
10. Medeiros RB, Papenfuss KJ, Hoium B, Coley K, Jadrich J, Goh SK, Elayaperumal A, Herrera JE, Resnik E, and Ni HT. Novel sequential ChIP and simplified basic ChIP protocols for promoter co-occupancy and target gene identification in human embryonic stem cells. *BMC Biotechnol.* 2009;9(59.

11. Geisberg JV, and Struhl K. Quantitative sequential chromatin immunoprecipitation, a method for analyzing co-occupancy of proteins at genomic regions in vivo. *Nucleic Acids Res.* 2004;32(19):e151.
12. Tauseef M, Knezevic N, Chava KR, Smith M, Sukriti S, Gianaris N, Obukhov AG, Vogel SM, Schraufnagel DE, Dietrich A, et al. TLR4 activation of TRPC6-dependent calcium signaling mediates endotoxin-induced lung vascular permeability and inflammation. *J Exp Med.* 2012;209(11):1953-68.

Supplementary Table 1

List of mouse and human primers used in this study.

Mouse Primer

Gene	Forward	Reverse
Erg	GGAGTGGGCGGTGAAAGA	AAGGATGTCGGCGTTGTAGC
Fli1	ACTTGGCCAAATGGACGGACTAT	CCCGTAGTCAGGACTCCCG
Cxcr2	TCACAAACAGCGTCGTAGA	GACAGCATCTGGCAGAATAG
Il1β	ACTACAGGCTCCGAGATGAA	TGGGTCCGACAGCACGAGGC
Tnfα	CCCCAAAGGGATGAGAAGTT	ACTTGGTGGTTTGCTACGA
Icam1	CAATTTCTCATGCCGCACAG	AGCTGGAAGATCGAAAGTCCG
Nlrp3	AGAAGAGACCACGGCAGAAG	CCTTGGACCAGGTTCAGTGT
Mip2α	CAG AAT TCA CTT CAG CCT AGC GCC AT	GCT CTA GAG TCA GTT AGC CTT GCC TTT G
Il6	AGTCCGGAGAGGAGACTTCA	TTGCCATTGCACAACTCTTT
Cd47	GTCTCCTCTGACTTCAACAGCG	ACCACCCTGTTGCTGTAGCCA
A20	CCGGCCCCCAGGTTCCAGA	AGGCCCGGGCACATTTCAGC
Gapdh	AAGGTCATCCCAGAGCTGAA	CTGCTTCACCACCTTCTTGA
β-actin	CCAGAGCAAGAGAGGTATCC	CTGTGGTGGTGAAGCTGTAG
Ubc	CCCAGTGTTACCACCAAGAAG	CCCCATCACACCCAAGAACA

Human Primer

Gene	Forward	Reverse
ERG	CTTCAACCCTCAGGCGGACA	GGAAAAGCGGCCAGTATAGGT
FLI1	AACGCCAGCTGTATCACCTG	AGCATCCAGTAAGTGTGCAG
IL8	ATGACTTCCAAGCTGGCCGTGGCT	TCTCAGCCCTCTTCAAAAACTTCTC
A20	AAAGCCCTCATCGACAGAAA	CAGTTGCCAGCGGAA TTTA
CXCR2	CAG TTA CAG CTC TAC CCT GCC	CCA GGA GCA AGG ACA GAC CCC
CYLD	TCAGGCTTATGGAGCCAAGAA	ACTTCCCTTCGGTACTTTAAGGA
USP9X	AAGTGAAGCATGTCAGCGATT	GCCACACATAGCTCCACCA
USP18	AGACCTGCTGCCTTAACTCC	GGACGCTTCTCCTCTGCTC
IL6	GTAGCCGCCCCACACAGA	CATGTCTCCTTTCTCAGGGCTG
NLRP3	CGTGAGTCCCATTAAGATGGAGT	CCCGACAGTGGATATAGAACAGA
GAPDH	GTCTCCTCTGACTTCAACAGCG	ACCACCCTGTTGCTGTAGCCAA
β-ACTIN	TGACAGGATCGAGAAGGAGA	CGCTCAGGAGGAGCAATG

Supplementary Table 2

List of primers used in Sequential Chromatin Immunoprecipitation Assay.

Gene	Forward	Reverse
Erg	TTTGGAGACCCGAGGAAAGC	CTGTTCAGAACCTGACGGCT
NFκB	CGCCACCCGGCTTCAGAAT	TATGGGCCATCTGTTGGCAG
