# Supplementary material for: ERG-deficient endothelium identifies IL-8/CXCR2 axis as a therapeutic target for resolving neutrophilic lung vascular injury
Source: JCI Insight. 2026 Mar 5;11(7):e195989. doi: 10.1172/jci.insight.195989 (PMC13134714; doi:10.1172/jci.insight.195989)
Supplement: Unedited blot and gel images [file jciinsight-11-195989-s280.pdf]

Figure 1C

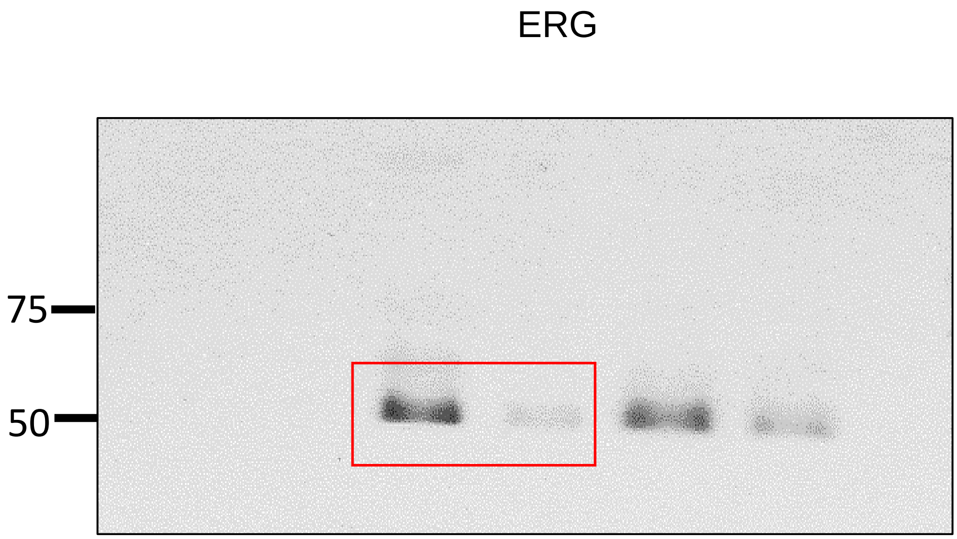

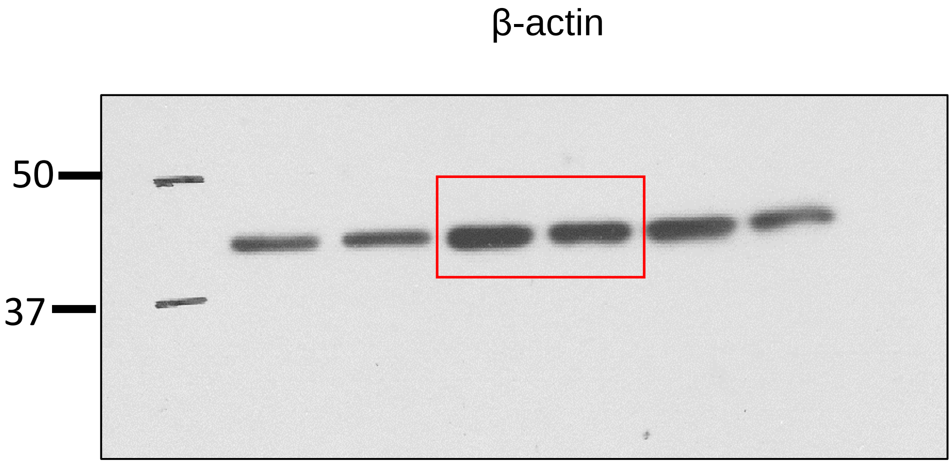

Figure 5B

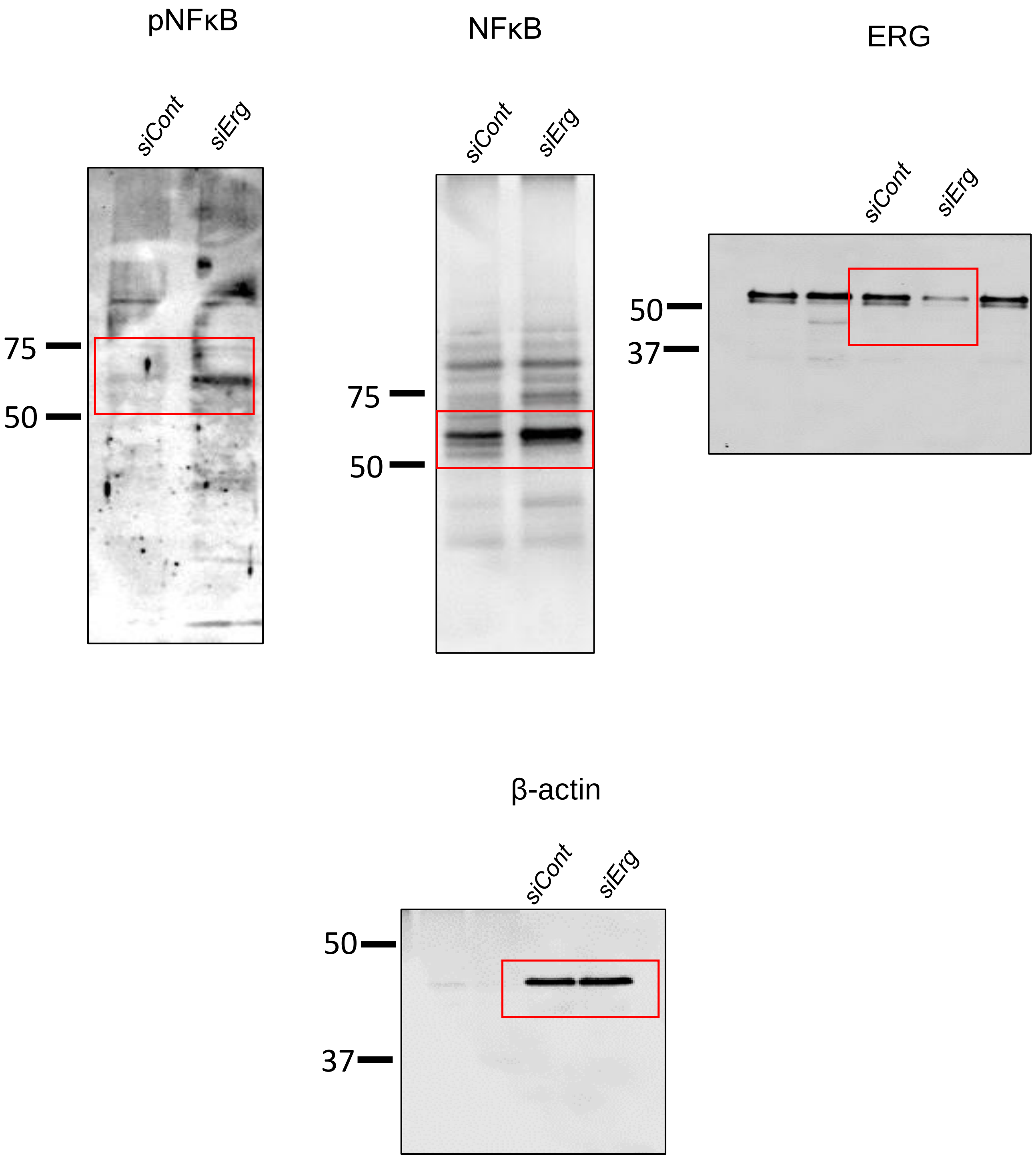
pNFκB
siCont
siErg
75
50
NFκB
siCont
siErg
75
50
ERG
siCont
siErg
50
37
β-actin
siCont
siErg
50
37

Figure 5 B

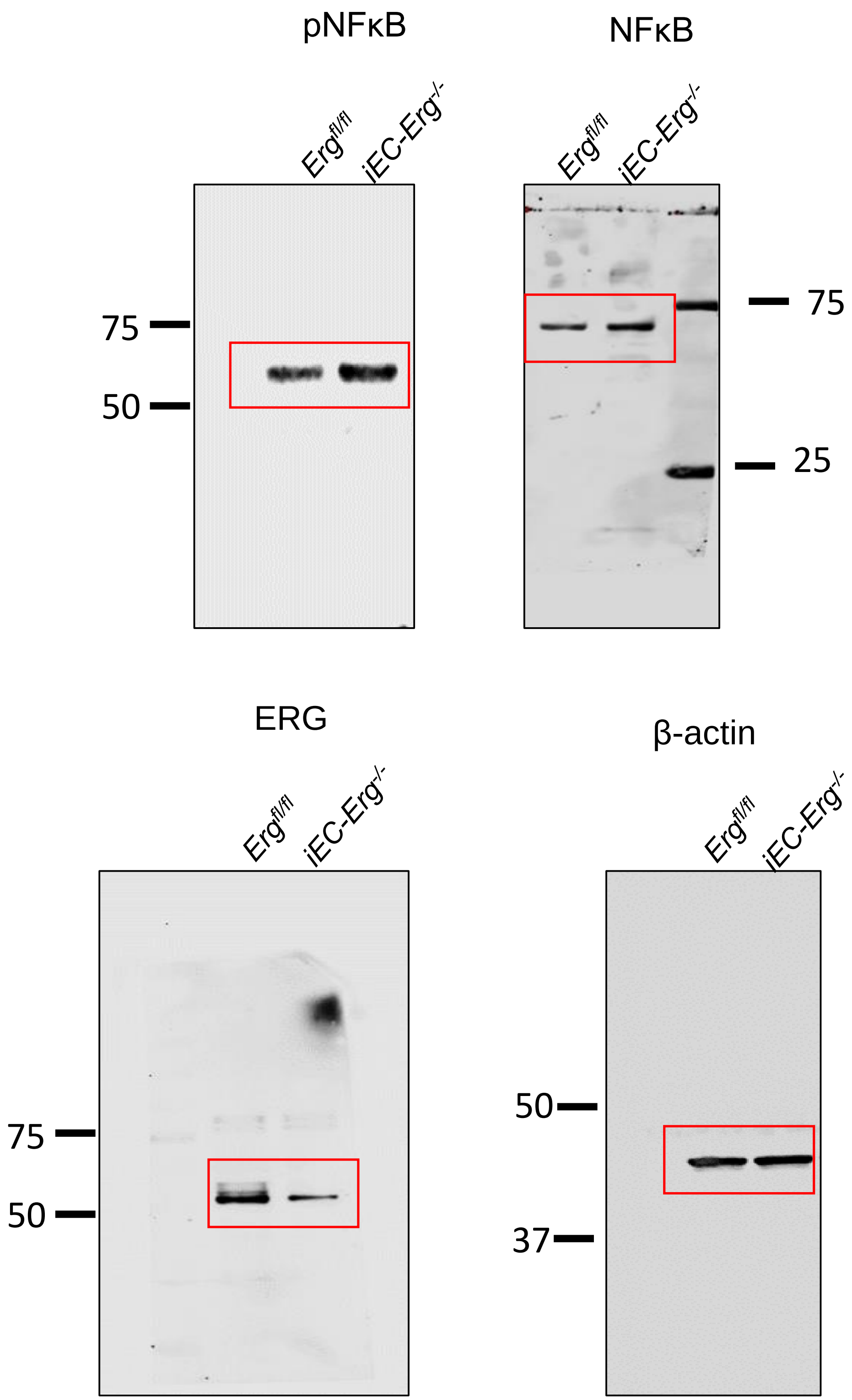

pNFκB
Erg fl/fl
iEC-Erg -/-
75
50
NFκB
Erg fl/fl
iEC-Erg -/-
75
25
ERG
Erg fl/fl
iEC-Erg -/-
75
50
β-actin
Erg fl/fl
iEC-Erg -/-
50
37

Figure 5B

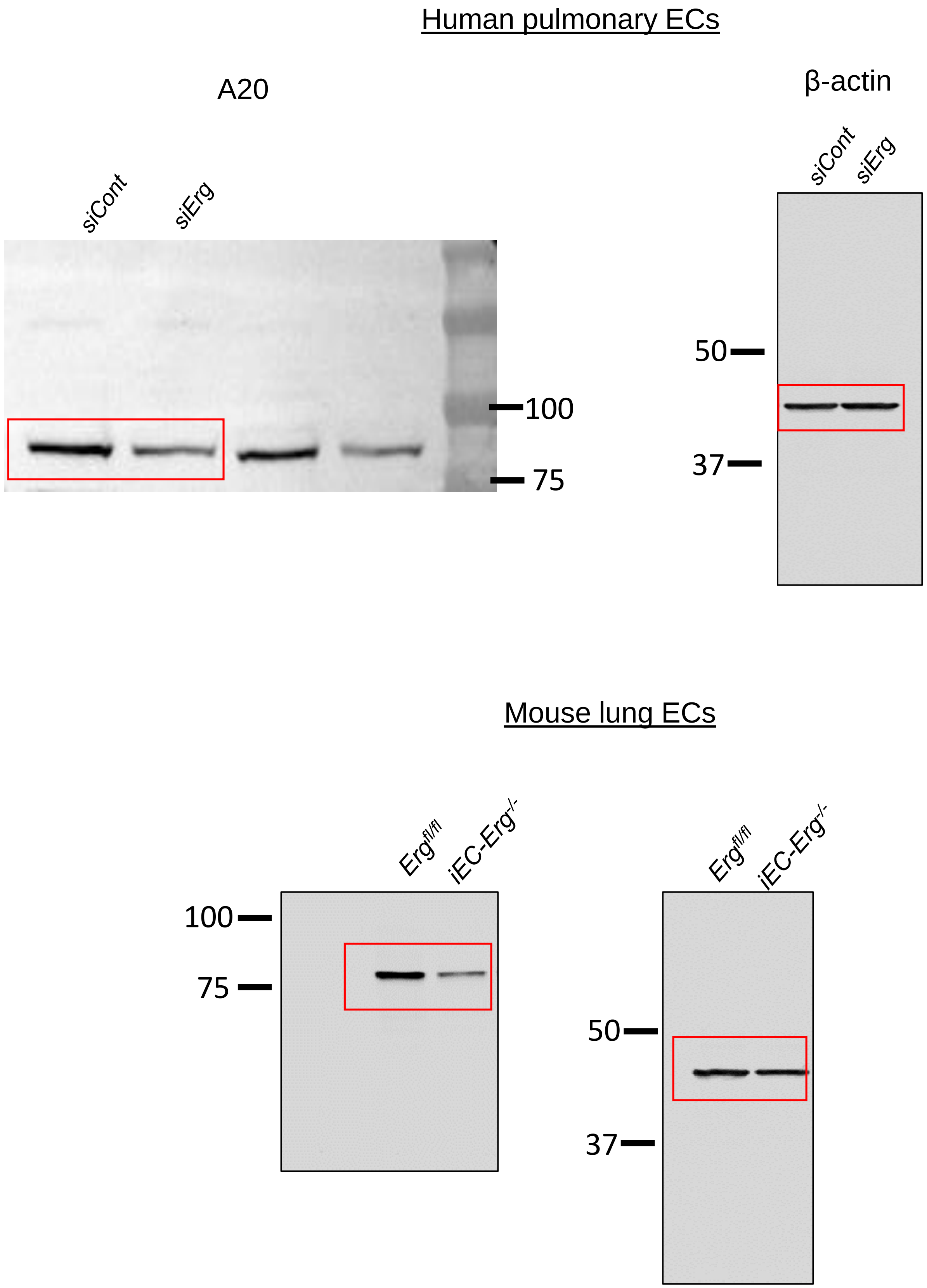
Human pulmonary ECs
A20
β-actin
siCont
siErg
100
75
50
37
Mouse lung ECs
Erg fl/fl
iEC-Erg -/-
100
75
50
37

Figure 7C

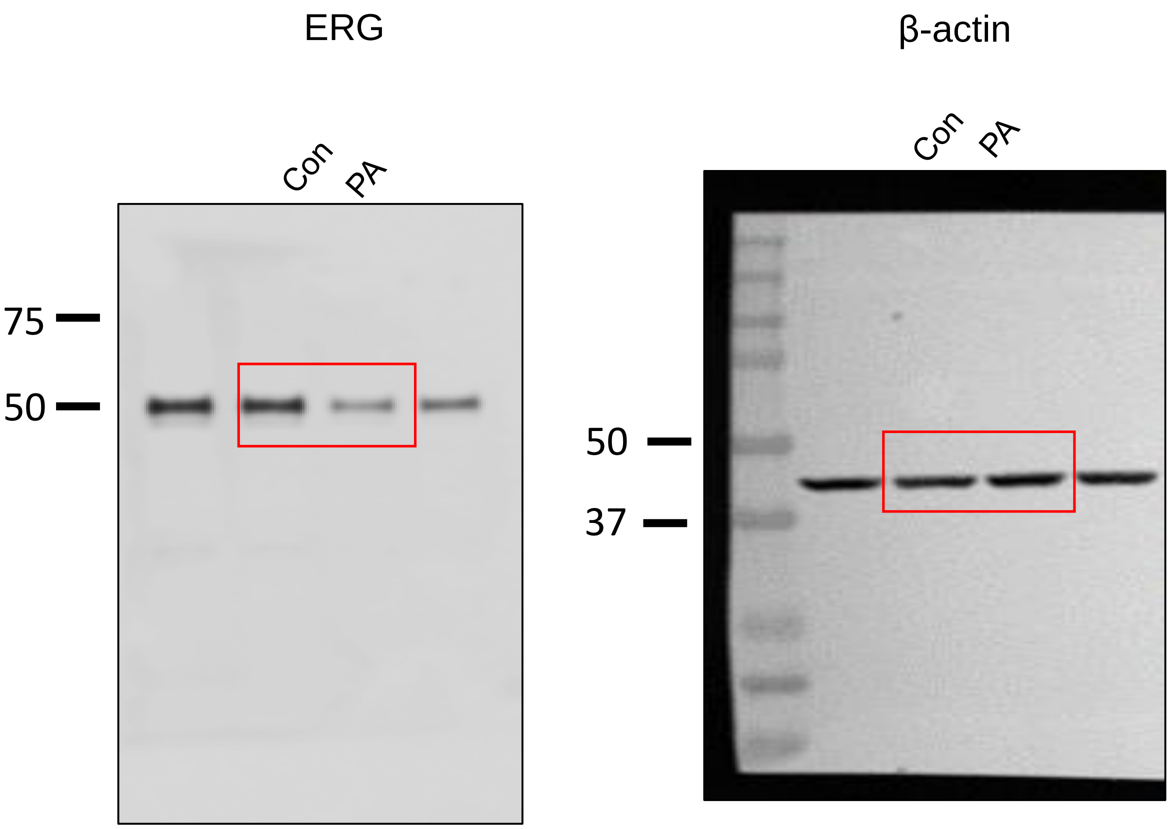

Figure 5I

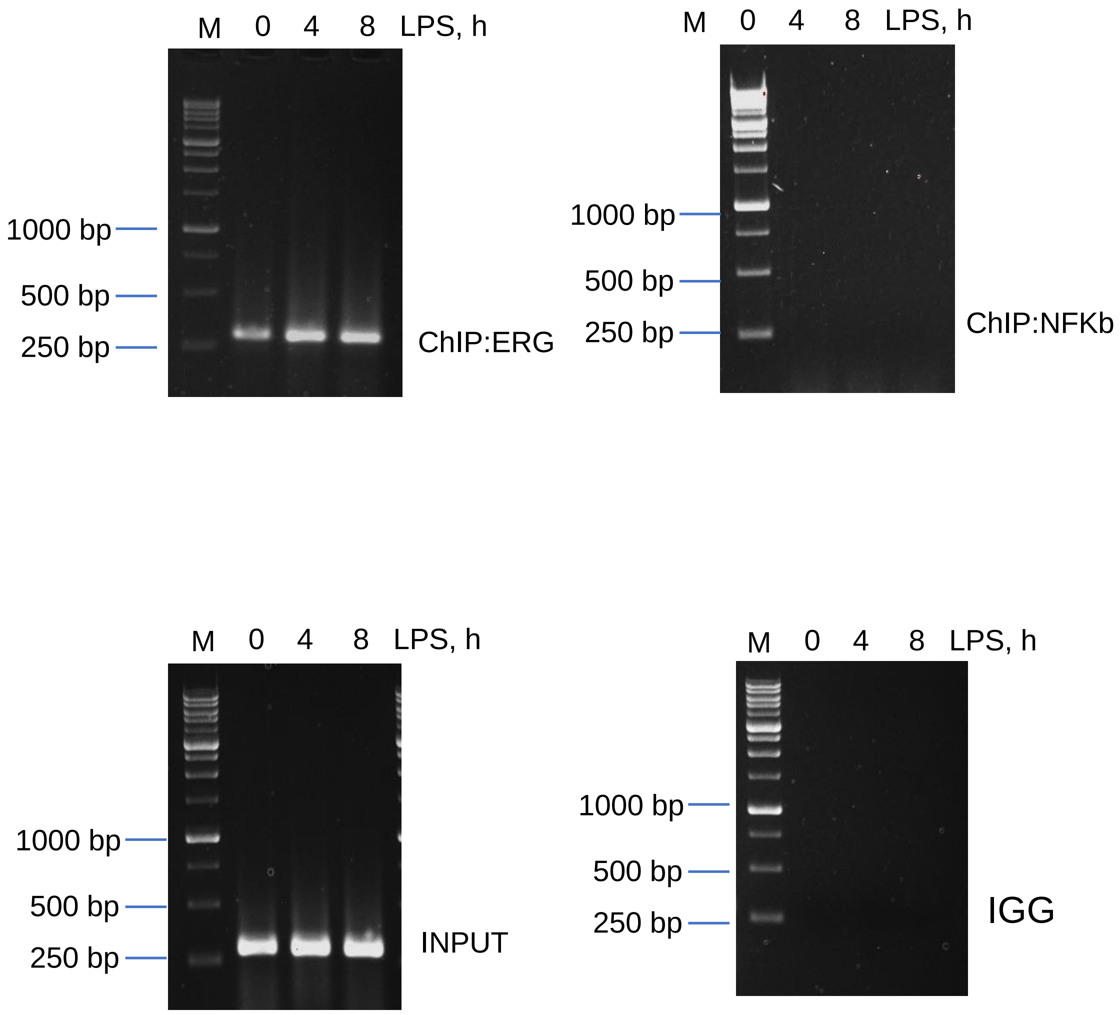
M 0 4 8 LPS, h
1000 bp
500 bp
250 bp
ChIP:ERG
M 0 4 8 LPS, h
1000 bp
500 bp
250 bp
ChIP:NFKb
M 0 4 8 LPS, h
1000 bp
500 bp
250 bp
INPUT
M 0 4 8 LPS, h
1000 bp
500 bp
250 bp
IGG

Figure 5G

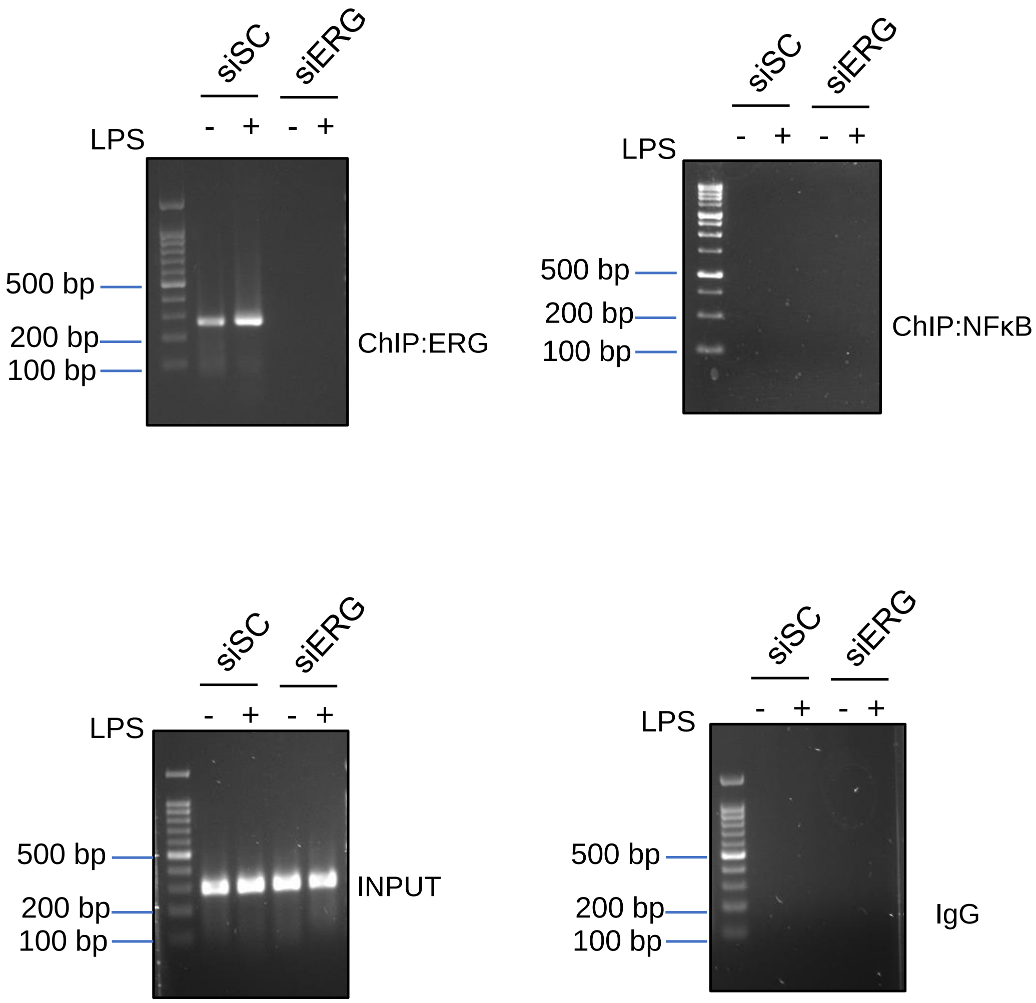

siSC
siERG
LPS
- + - +
500 bp
200 bp
100 bp
ChIP:ERG
siSC
siERG
LPS
- + - +
500 bp
200 bp
100 bp
ChIP:NFκB
siSC
siERG
LPS
- + - +
500 bp
200 bp
100 bp
INPUT
siSC
siERG
LPS
- + - +
500 bp
200 bp
100 bp
IgG
